# Morphological transformation enhances Tumor Retention by Regulating the Self-assembly of Doxorubicin-peptide Conjugates

**DOI:** 10.7150/thno.45088

**Published:** 2020-07-09

**Authors:** Liu Xu, Yutong Wang, Chenqi Zhu, Shujing Ren, Yurou Shao, Li Wu, Weidong Li, Xiaobin Jia, Rongfeng Hu, Rui Chen, Zhipeng Chen

**Affiliations:** 1 College of pharmacy, Nanjing University of Chinese Medicine, Nanjing 210023, China.; 2 Department of Pharmacy, the Affiliated Suzhou Hospital of Nanjing Medical University, Suzhou 215002, China.; 3 School of Traditional Chinese Pharmacy, China Pharmaceutical University, Nanjing 211198, China.; 4 Key Laboratory of Xin'an Medicine, Ministry of Education, Anhui University of Chinese Medicine, Hefei, Anhui 230038, People's Republic of China.

**Keywords:** intravenous nanomedicine, acid-responsive, morphology transformation, long retention, peptide-drug conjugates

## Abstract

**Rationale:** Both spatial accuracy and temporal persistence are crucial in drug delivery, especially for anti-tumor intravenous nanomedicines, which have limited persistence due to their small particle sizes and easy removal from tumors. The present study takes advantage of morphological transformation strategy to regulate intravenous nanomedicines to display different sizes in different areas, achieving high efficient enrichment and long retention in lesions.

**Methods:** We designed and synthesized functional doxorubicin-peptide conjugate nanoparticles (FDPC-NPs) consisting of self-assembled doxorubicin-peptide conjugates (DPCs) and an acidic-responsive shielding layer named the functional polylysine graft (FPG), which can regulate the assembly morphology of the DPCs from spherical DPC nanoparticles (DPC-NPs) to DPC-nanofibers (DPC-NFs) by preventing the assembly force from π-π stacking and hydrogen bond between the DPC-NPs. The morphology transformation and particle changes of FDPC-NPs in different environments were determined with DLS, TEM and SEM. We used FRET to explore the enhanced retention effect of FDPC-NPs in tumor site *in vivo*. HPLC-MS/MS analytical method was established to analyze the biodistribution of FDPC-NPs in H22 hepatoma xenograft mouse model. Finally, the antitumor effect and safety of FDPC-NPs was evaluated.

**Results:** The FDPC-NPs were stable in blood circulation and responsively self-assembled into DPC-NFs when the FDPC-NPs underwent the acid-sensitive separation of the shielding layer in a mildly acidic microenvironment. The FDPC-NPs maintained a uniform spherical size of 80 nm and exhibited good morphological stability in neutral aqueous solution (pH 7.4) but aggregated into a long necklace-like chain structure or a crosslinked fiber structure over time in a weakly acidic solution (pH 6.5). These acidity-triggered transformable FDPC-NPs prolonged the accumulation in tumor tissue for more than 5 days after a single injection and improved the relative uptake rate of doxorubicin in tumors 31-fold. As a result, FDPC-NPs exhibited a preferable anti-tumor efficacy and a reduced side effect *in vivo* compared with free DOX solution and DOX liposomes.

**Conclusions:** Morphology-transformable FDPC-NPs represent a promising therapeutic approach for prolonging the residence time of drugs at the target site to reduce side effect and enhance therapeutic efficacy. Our studies provide a new and simple idea for the design of long-term delivery systems for intravenous chemotherapeutic drugs.

## Introduction

Nanoparticles have garnered considerable attention in transporting drugs to tumor tissues with controlled drug release and improved bioactivity 1-3. Despite the great success of nanoparticles in tumor therapy, these nanoparticles still face the inherent restriction of small size and easy removal from tumors. Substantial efforts have been devoted to the study of drug targeting and enrichment in tumors. For example, Xuehai Yan reported supramolecular curcumin nanoagents via the amino acid coordination-driven self-assembly to simultaneously increase the biological stability and tumor accumulation of curcumin 4. Mingdong Huang developed a fusion protein assembly nanoparticle with cargo loading and tumor targeting capability 5. Guangjun Nie et al. exploited a naturally occurring protein as an intelligent, tumor-targeting, hydrophobic antitumor drug delivery molecular machine 4. Unfortunately, how to extend the residence time of the drugs in tumors after drug enrichment has rarely been reported. It is particularly important to find a strategy to lengthen the drug residence time while enriching the drug at the tumor. Therefore, increasing importance has been placed on the fact that intravenous nanomedicines not only highlight spatial accuracy but also temporal persistence.

Inspired by the long retention of large-scale structures in lesions, morphological transformation is an emerging strategy to achieve the long-term retention of tumors after intravenous injection, which is used for imaging and manufacturing artificial extracellular matrix for the inhibition of metastasis. For example, Hao Wang et al. reported an *in situ* bioinspired construction of artificial extracellular matrix that acted as a long-term barrier with a competing binding capability with natural extracellular matrix, resulting in the highly efficient inhibition of tumor metastasis and tumor growth 6. Gianneschi et al. exploited an enzyme-directed, nanoparticle accumulation and retention process to form a new, slow-clearing morphology for tumor molecular diagnostics 7. Few reports have applied this strategy to chemotherapeutic drugs to enhance the long-term retention in tumors. The key to the successful construction of a transformable drug delivery system (DDS) for intravenous injection can be summarized as follows: First, the stability of the drug in the process of morphological transformation must be ensured, and the drug can be slowly released in the lesion. Second, the DDS should be equipped with suitable surface potential 8-10, hydrophilicity 11-13, an appropriate size and morphology 14-16 to ensure good biocompatibility 17-19, and enough stability and circulation time in blood 20-22. Third, after reaching the tumor through the enhanced permeability and retention (EPR) effect 23, 24, the drug must be able to reside in the lesion.

**Scheme 1:**
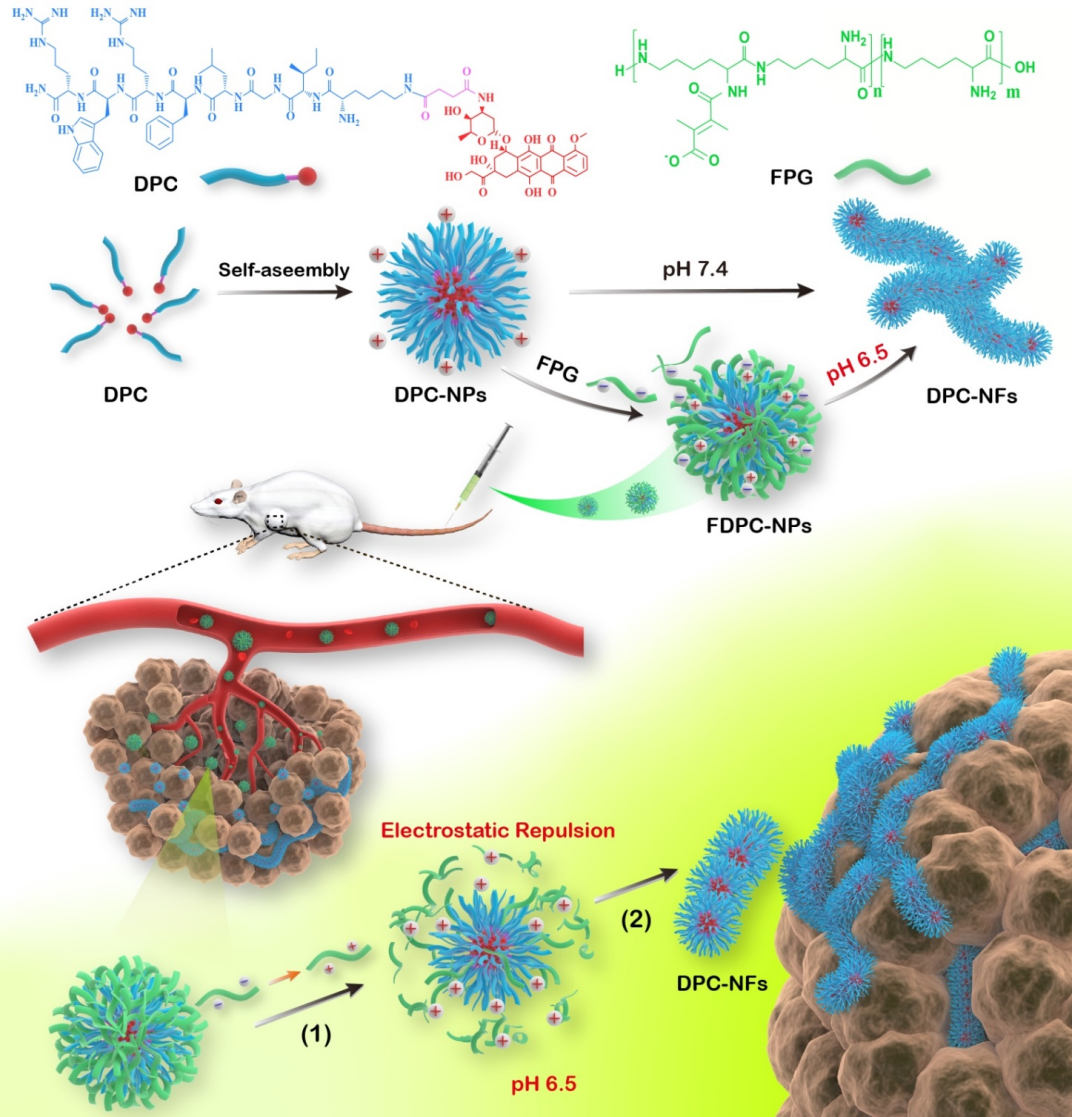
The self-assembly behavior of DPCs and the morphologic transformation of the acid-responsive FDPC-NPs *in vitro* and *in vivo*. The schematic shows the morphology transformation from NPs to NFs triggered by a change in pH after reaching the tumor site through the EPR: (1) The outer FPG converts to a positive charge, and separation of the core-shell structure occurs; and (2) The DPC-NPs further assemble into DPC-NFs driven by π-π stacking, leading to long-term drug retention in the tumor.

In this work, we reported doxorubicin-peptide conjugates (DPCs) with an extracellular tumor acid-responsive sphere-fiber transformation for enhanced residence in tumors. As illustrated in Scheme 1, the chemotherapy drug doxorubicin (DOX) was coupled with a peptide (KIGLFRWR) to design a DPC molecule with assembly ability. First, the DPCs, driven by hydrophobic forces from the hydrophobic drug DOX and the IGL fragment, can form spherical DPC nanoparticles (DPC-NPs) 25, 26. Then, along with hydrogen bond between peptides, the aromatic amino acids F and W give the DPC-NPs the ability of self-assembly to DPC-nanofibers (DPC-NFs) due to π-π stacking. The step-by-step assembly process provides opportunities for morphological transformation control. To meet the particle size requirements for intravenous injection, the acid-responsive material 2,3-dimethylmaleic anhydride grafted polylysine, named the functional polylysine graft (FPG), was designed as a shielding layer for DPC-NPs and formed functional doxorubicin-peptide conjugate nanoparticles (FDPC-NPs) by an electrostatic interaction to avoid π-π stacking interactions and hydrogen bond between the DPC-NPs. Therefore, the FDPC-NPs could maintain an appropriate size in blood vessels until entering the tumor stroma by the EPR effect. When the FDPC-NPs passed through the blood vessel and entered the weakly acidic microenvironment of the tumor, the surface potential of the shield was reversed from negative to positive because of acid-sensitive 2,3-dimethylmaleic groups on the FPG. Therefore, FPG would separate from the DPC-NPs because of the mutual repulsion effect from the like charges. Then, DPC-NPs self-assembled into DPC-NFs, thereby staying in the tumor region for a long time. After that, the fibers degraded gradually and free drug penetrated into tumor cells, exerting sustained anti-tumor effect. This study is original and provides new ideas for the design of targeted and long-acting drug delivery systems for tumor therapy.

## Methods

### Materials

9-Fluorenylmethyl (Fmoc)-amino acids and Rink Amide resin (100-200 mesh, substitution factor: 0.486 mM) were obtained from GL Biochem (Shanghai, China). Doxorubicin hydrochloride (DOX, >95%) was obtained from Aladdin (Shanghai, China). All other chemicals and reagents were of analytical or HPLC grade. The SMMC-7721 human hepatoma and H22 hepatoma cell lines were purchased from the National Infrastructure of Cell Line Resource. ICR mice were purchased from the Experimental Animal Center at Nanjing University of Chinese Medicine, China.

### Synthesis and structural confirmation of DPC

DPC was synthesized through a three-step reaction as illustrated in Scheme S1. Briefly, the peptide (KIGLFRWR) was first synthesized according to standard solid-phase Fmoc peptide synthesis techniques. In the last coupling step, Fmoc-Lys(Dde)OH was used to produce Fmoc-peptides. Hydrazine (2%) in anhydrous N,N'-dimethylformamide (DMF) was used for the deprotection of the Dde group, 3 times for 5 min each time. Then, succinic acid was added to the side chain of Lys for further reaction. A ninhydrin test was performed to test for free amines in the reactions. The finished peptide was cleaved from the AM resin using a mixture of trifluoroacetic acid (TFA): triisopropylsilane (TIS): purified water at a ratio of 95:2.5:2.5 and shaken for 2 h at room temperature. The mixture was filtered into cold diethyl ether, the ether and precipitated peptide were centrifuged at 10000 rpm for 5 min to obtain the crude peptide, and the crude peptide was washed with diethyl ether 3 times. The ether was decanted, and the peptide was dried under vacuum. The crude materials were dissolved in methanol and purified by preparative reversed phase high performance liquid chromatography (RP-HPLC). The fractions containing the desired product were lyophilized. The drug DOX was then manually coupled to the carboxyl group of succinic acid, followed by deprotection of the Fmoc group to produce the DPC. MALDI-TOF-MS was used to confirm the product.

### Self-assemble mechanism study of DPCs

A total of 1.7 mg of DPCs was dissolved in 4 mL of purified water to prepare a self-assembled DPC solution. Hydrochloric acid (0.1 M) and a sodium hydroxide solution were used to adjust the pH to neutral (pH = 7.4). Then, a UV spectrophotometer was used to scan and record the changes in the UV absorption spectrum of the DPC solution at different time points.

1.05 mg of DPCs was dissolved into (1) 5 mL of urea solutions with different concentration (0.5 mM, 5 mM, 50 mM, pH 7.0) respectively; (2) 5 mL of hydrochloric acid or sodium hydroxide solution with different pH values (pH 4.0, 6.0, 8.0) respectively. Particle sizes and zeta potential were measured by dynamic light scattering (DLS) at 0.5, 1, 3, 6 and 12 h. After that, 10 μL of the sample was removed at each time point to prepare TEM samples immediately. The sample solutions (10 µL) were spotted on carbon-coated copper grids, dried for 10 min, and then negatively stained with 5% uranyl acetate. Samples were analyzed by a Hitachi transmission electron microscope.

### Synthesis of FPG

FPG is a kind of ε-PL graft modified by DMMA. To an 8 mL purified water solution containing 400 mg of ε-PL and 360 mg of N-hydroxysuccinimide (NHS), 360 mg of DMMA in 10 mL of purified water was added. Next, 500 mg of 1-ethyl-3-(3-dimethylaminopropyl)carbodiimide (EDCI) was added to the mixture above, and the obtained aqueous solution was alkalinized to approximately pH 8.5 with triethylamine. The reaction mixture was stirred at room temperature for approximately 12 h. Then, dialysis was performed with purified water as the dialysis medium to purify the reaction solution. The purified product was lyophilized. MALDI-TOF-MS and C-NMR spectrum were used to confirm the structure.

### Preparation of FDPC-NPs

A total of 1.7 mg of DPCs was dissolved in 2 mL of purified water. Then, 6.5 mg of FPG was added to the solution. Hydrochloric acid (0.1 M) and a sodium hydroxide solution were used to adjust the pH to neutral (pH = 7.4). The solution was incubated at room temperature to obtain the FDPC-NPs (500 μM).

### Characterization of DPCs and FDPC-NPs

A total of 5.0 mg of DPCs was dissolved in 12 mL of purified water, and the solution was immediately equally divided into 3 portions of 4 mL each. The pH of the first portion was adjusted to 7.4 and the second to 6.5 with 0.1 M sodium hydroxide solution and hydrochloric acid. A total of 6.5 mg of FPG was added to the third portion to obtain a self-assembly solution of FDPC-NPs, and 0.1 M hydrochloric acid and a sodium hydroxide solution were used to adjust the pH to 7.4. DLS was performed to measure the particle size and distribution of the different solutions and record the results at different time points. After 6 h, the self-assembled solution of FDPC-NPs was divided into 2 portions of 2 mL each: one portion was adjusted to pH 6.5 to mimic the pH change when nanoparticles entered the tumor, and another portion was maintained at pH 7.4. As control, 6.5 mg of FPG was dissolved in 4 mL of purified water, and the solution was immediately equally divided into 2 portions of 2 mL each. One portion was adjusted to pH 7.4 with 0.1 M sodium hydroxide solution and another portion was adjusted to pH 6.5 with hydrochloric acid. The particle size of each group of solutions was measured at different time points. After each DLS measurement, 10 μL of the sample was removed at each time point to prepare TEM samples to observe the assembled morphology using the method mentioned above.

### Self-assembly of FPDC-NPs in cell culture

SMMC7721 cells (5×10^4^ cell/well) were seeded on a 48-well plate with glass coverslips for 24 h, then wash the cells with PBS and incubated them with 200 μL of DMEM, DOX solution (50 μM) and FPDC-NPs (50 μM). After 12 h incubation, pH value of cell culture was measured. The cells were washed with PBS, followed by solidification and dehydration process. Finally, the treated cells were placed in a vacuum drying oven overnight. Samples were analyzed by a scanning electron microscope (SEM) (Hitachi SU8010).

### Stability study of FDPC-NPs

FDPC-NPs (0.1mM, calculated as DOX) were dissolved in PBS (pH7.4). After 50-fold dilution with PBS (pH7.4), particle sizes were measured at 0.25, 1, 3, 6, 12 h by DLS, and 10 μL of the samples were removed at each time point. TEM and DLS were used to evaluate their particle sizes and morphologies. To mimic intravascular microenvironment, FDPC-NPs (1 mM, calculated as DOX) or DOX (1 mM) was co-incubation with 2 mL of rat plasma in dialysis bag (Mw: 2000 Da) in 8mL of PBS (pH7.4) at 37 °C. At designated time points* i.e.* 0.5, 1, 2, 4, 6, 8 and 12 h, samples were removed from the incubator, centrifuged for 10 min at 3000 rpm and filtered. The amount of DOX was quantified using high performance chromatography (HPLC) (Shimadzu, Japan). Chromatographic study was performed at 30 °C on a reversed-phase column (Kromasil 100-5-C 18, AKZO NOBEL, Sweden). The mobile phase consisted of acetonitrile and 0.1% trifluoroacetic acid (TFA), and a gradient method was employed for the analysis (20-70% acetonitrile over 0-25 min). Detection was performed at a wavelength of 480 nm (Ultra Violet detector, Shimadzu, Japan). Specificity, linearity, recovery, precision, accuracy and solution stability were performed for validation.

### *In vitro* release profile detection of FDPC-NPs

For* in vitro* release of DOX from FPCD-NPs, 1 mL of FDPC-NPs (1 mM, calculated as the DOX) and 1mL of DOX solution were placed in dialysis bag (Mw: 2000 Da) respectively. 10 mL of PBS (pH 7.4) and acidic PBS (pH 6.5) were used as dissolution medium (according to the requirements of the sump conditions) at 37 °C in a constant-temperature shaking tank. At specific times during incubation (*i.e.* 0.25, 0.5, 1, 3, 6, 12, 24, 48, 72, 96, 120, 144 and 168 h), 200 µL of supernatant was removed from the beaker, and the same volume of fresh PBS was replaced. All samples were analyzed by HPLC method metioned above. The cumulative release rates of DPCs and DOX were calculated according to the cumulative release rate calculation formula. All tests were performed in triplicate.


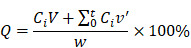


Where Q is the cumulative release rate, V is the volume of the released medium, C*_i_* is the concentration of the drug sampled at each time point, v' is the sample volume, and W is the total dose.

### Labeling the peptide and FPG with the fluorescent agent

In a typical fluorescence resonance energy transfer (FRET) experiment, the donor fluorescein and the acceptor fluorescein were first labeled on the two molecules. The FITC- and Cy5.5-labeled peptides were synthesized as follows. Briefly, 30 mg of peptide was dissolved in 15 mL of NaCO_3_-NaHCO_3_ buffer or 10 mL of dimethyl sulfoxide (DMSO). Then, 12 mg of FITC or 3 mg of Cy5.5-NHS was added to the solution with stirring. Then, the reaction mixture stirred at room temperature for 6 h in the dark. After the reaction, dialysis (M_w_: 1000 Da) was performed to remove the free FITC or Cy5.5. The purified reaction solution was lyophilized to obtain the FITC- or Cy5.5-labeled peptide.

The TRITC- and Cy7-labeled peptides were synthesized as follows. Briefly, 90 mg of FPG was dissolved in 30 mL of NaCO_3_-NaHCO_3_ buffer or 20 mL of DMSO. Then, 30 mg of TRITC or 6 mg of Cy7-NHS was added to the solution with stirring. Then, the reaction mixture stirred at room temperature for 6 h in the dark. After the reaction, dialysis (M_w_: 1000 Da) was performed to remove the unreacted TRITC or Cy7-NHS. The purified reaction solution was lyophilized to obtain TRITC or Cy7-labeled FPG.

### Preparation of the FRET-labeled nanoparticles

A total of 0.8 mg of FITC- or Cy5.5-labeled peptide was dissolved in 2 mL of purified water. Then, different amounts of TRITC- or Cy7-labeled FPG were added to the solution. Hydrochloric acid (0.1 M) and a sodium hydroxide solution (0.1 M) were used to adjust the pH to neutral (pH = 7.4). The solution was incubated at room temperature to obtain a solution of FRET-NPs. FITC and TRITC are a pair of FRET dyes used for *in vitro* studies, while Cy5.5 and Cy7 are a pair of FRET dyes used for* in vivo* imaging studies.

### Fluorescence measurements of the FRET-NPs

First, the fluorescence spectra of the dyes were detected and paired according to the spectral overlaps between the absorption (acceptors) and emission spectra (donors). The concentration of FITC (donor)-labeled peptide was maintained at 100 μM for all measurements. During the preparation of the FRET-NPs, different amounts of TRITC (acceptor)-labeled FPG were added to a solution of FITC-labeled peptide, and 0.1 M hydrochloric acid and 0.1 M sodium hydroxide solution were used to adjust the pH to 7.4 or 6.5; then, FRET fluorescence measurements were performed using a PerkinElmer LS 55 fluorescence spectrophotometer.

### Characterization of the FRET-NPs

A total of 1.5 mg of FITC-labeled peptide was dissolved in 10 mL of purified water to prepare a self-assembling solution of FITC-labeled peptide, and the solution was immediately divided into 2 portions of 5 mL each. A total of 1.5 mg of FPG mixed with 6 mg of TRITC-labeled FPG was added to the first portion to obtain a solution of FRET-NPs, and the other portion was kept as a control, to which 0.1 M hydrochloric acid and a sodium hydroxide solution were added to adjust the pH to 7.4. After 4 h, the FRET-NPs were divided into 2 portions of 2.5 mL each; one portion was adjusted to pH 6.5, and the other portion was maintained at pH 7.4. The particle size of each group of solutions was measured at given time points *i.e.* 1, 2, 4, 6, 8 and 12 h. After each DLS measurement, 10 μL of sample was removed at each time point to prepare TEM samples to observe the assembled morphology by the TEM method mentioned above.

### Near-infrared fluorescence (NIRF) imaging *in vivo*

An H22 hepatoma xenograft mouse model was utilized in this study. Mice were inoculated with 2.0×10^6^ H22 cells, and studies were carried out once the tumors reached approximately 1000 mm^3^ in volume. All animal procedures were approved by the Institutional Animal Care and Use Committee of Nanjing University of Chinese Medicine. A total of 1.0 mg of Cy5.5-labeled peptide was dissolved in 1 mL of saline, and then 1.5 mg of Cy7-labeled FPG mixed with 6.0 mg of FPG was added. The solution was incubated at room temperature to obtain a solution of FRET-labeled nanoparticles. Cy5.5-labeled liposomes were prepared as a control. For optical imaging, animals were anesthetized with isoflurane at a maintenance dose of 1.5% in an oxygen gas stream. Two groups of H22 hepatoma mice were intravenously injected with 0.2 mL of FRET-NPs or Cy5.5-labeled liposomes. NIRF imaging was performed using a PE IVIS Spectrum imaging system for each group of mice at selected postinjection time points* i.e.* 0.5, 1, 2, 4, 6, 8, 10, 12, 18, 24, 36, 48, 60, 72 and 96 h. Three optical channels were recorded with selected excitation and emission bandpass filters 27: Cy5.5 (λ_ex_=640 nm and λ_em_=720 nm); Cy7 (λ_ex_ =745 and λ_em_ = 800 nm); and FRET (λ_ex_=640 nm and λ_em_= 800 nm). The exposure time for each image was 0.1s.

### Tissue distribution in the tumor mouse model

An H22 hepatoma xenograft mouse model was utilized in this study. Mice were inoculated with 2.0×10^6^ H22 cells, and studies were carried out once the tumors reached approximately 1000 mm^3^ in volume. All animal procedures were approved by the Institutional Animal Care and Use Committee of Nanjing University of Chinese Medicine. Two groups of ICR mice bearing ectopic H22 tumors (n=6) were injected with either FDPC-NPs at 7.2 mg of DOX equivalent or DOX solution per kg through the tail vein. At the given time points, the animals were sacrificed. Tumor, liver, spleen, lung, kidney, heart and brain samples were collected and weighed. Saline was added to the samples at a ratio of 1:2, and the samples were cut into small pieces by an automatic homogenizer. The homogenate from each group was separated into 90 μL portions in 2 mL centrifuge tubes, 10 μL of internal standard solution was added, vortexing was performed for 10 min, 500 μL of acetonitrile was added, vortexing and mixing were performed for 10 min, and the samples were centrifuged (13 000 r min^-1^) for 5 min. The supernatant was transferred to 1.5 mL tubes and centrifuged (13 000 r min^-1^) for 5 min at high speed. The supernatant (200 μL) was used for ultra-performance liquid chromatography (UPLC)-MS/MS analysis 28.

### MTT assay

SMMC-7721 cells (2×10^4^ cells/well) or LO2 cells (2×10^4^ cells/well) were incubated in 96-well plates for 24 h and then incubated with different concentrations of FDPC-NPs, DOX, peptide or FPG for 24 h. After incubation, 200 µL/well MTT solution (0.5 mg/mL in DMEM) was added followed by incubation for 4 h. Then, the culture medium was removed and replaced with 150 µL/well DMSO. The color intensity of the DMSO solution was measured at 490 nm using a microplate spectrophotometer to calculate the cell viability.

### Flow cytometry analyses

SMMC-7721 cells (2×10^4^ cells/well) were incubated in 96-well plates for 24 h and then incubated with different concentrations of FDPC, DOX, peptide or FPG for 24 h. After incubation, the culture was aspirated, and each well was washed 3 times with PBS. Then, 200 μL of different concentrations of DOX or FDPC-NPs was added to high-sugar DMEM as the medium. After incubation for 24 h, the drug-containing medium in the upper layer of each well was carefully pipetted into a 4 mL centrifuge tube. Each well was digested with 0.5 mL of trypsin without ethylenediaminetetraacetic acid (EDTA) for 4 min, and then 0.5 mL of DMEM containing 10% fetal bovine serum was added to prevent excessive digestion. The cells in each well were gently pipetted, collected and combined with the corresponding drug-containing supernatant medium. The combined solution was centrifuged at 2000 r min^-1^ for 5 min. After that, the supernatant was carefully aspirated, and the cells were washed once with ice-cold PBS. The collected cells were resuspended in 195 μL of annexin-V working solution and then stained with 5 μL of FITC. After 5 min at room temperature in the dark, 10 μL of propidium iodide (PI, 1.21 mg/mL in Tris) was added and further incubated for 10 min in darkness. Cells without any treatment were used as the control group. The apoptosis rate was analyzed using a BD FACS Calibur flow cytometer, and for each experiment, over 10,000 events per sample were recorded.

### Therapeutic treatment and inhibition of tumor growth

Tumor progression and body weight of ICR mice (6-8 weeks old) bearing H22 hepatoma subcutaneous xenografts were monitored every 2 days. The length (maximum diameter, L) and width (minimum diameter, W) of the tumor were measured with digital calipers. The tumor volume was calculated using the following formula: V = LW^2^/2. When the average tumor volume reached approximately 100 mm^3^, the mice were randomly divided into four groups (n=6) and treated via the tail vein with saline control, DOX solution, DOX liposomes or FDPC-NPs at 10 mg DOX equivalent per kg. The dosing frequency was once every three days. Tumor volumes and body weights were measured every two days. All of the mice were sacrificed and dissected in the 13^th^ day. Then the tissues were washed with cold PBS, weighed and photographed. Histological sections of the organic tissues were stained with H&E.

### Data analysis

Statistical analyses were performed using Statistical Package for the Social Sciences (SPSS) version 22.0 and GraphPad Prism version 6.0. Statistical comparison was carried out via Student's unpaired t-test or two-way analysis of variance (ANOVA). Significant and very significant differences are shown by *P < 0.05, **P < 0.01 and ***P < 0.001, respectively.

## Results and Discussion

### DPC and FPG were synthesized and verified

As two components of the FDPC-NPs, DPC and FPG were first synthesized. A detailed preparation approach for DPC is illustrated in Figure S1 in the Supporting Information. The peptide (KIGLFRWR) was prepared by solid-phase peptide synthesis (SPPS) approach. The molecular weight of peptide was confirmed with MS (Figure S2), and the peak at m/z 1075.34 represented the peptide (Mw = 1074.35 Da). Results from HPLC chromatograms showed the purity of the peptide was 95.1% (Figure S3). The molecular weight of DPC was confirmed with matrix-assisted laser desorption/ionization time-of-flight mass spectrometry (MALDI-TOF-MS). As shown in Figure S4, the peak at m/z 1699.2974 represented DPC (Mw = 1699.84 Da), and the detachment of partial DOX from DPC during the detection process induced the appearance of the peak at m/z 1303.3483. The purity of DPC was 96.4% (Figure S5). FPG was synthesized by modifying polylysine (ε-PL) with 2,3-dimethylmaleic anhydride (DMMA) (Figure S6) 29. With C-NMR spectrum, we confirmed its chemistry structure (Figure S7). ε-PL is a mixture composed of a variable number of polymerized lysine which displayed a series of peaks in the MALDI-TOF-MS spectrum (Figure S8A), and molecular weight distribution of FPG was examined with MALDI-TOF-MS (Figure S8B). Structure of FPG was also confirmed with C-NMR (Figure S9). The peak at 173.44 ppm represents the carbon signal of the amino linkage between ε-PL and side chain; the peak at 138.22 ppm represents the carbon signal of C-C double bond in the side chain of FPG; and peaks at 16.04 ppm and 8.11 ppm represent the carbon signal of CH_3_ in the side chain of FPG.

### DPCs self-assembly process is driven by π-π stacking and hydrogen bonding

**Figure 1:**
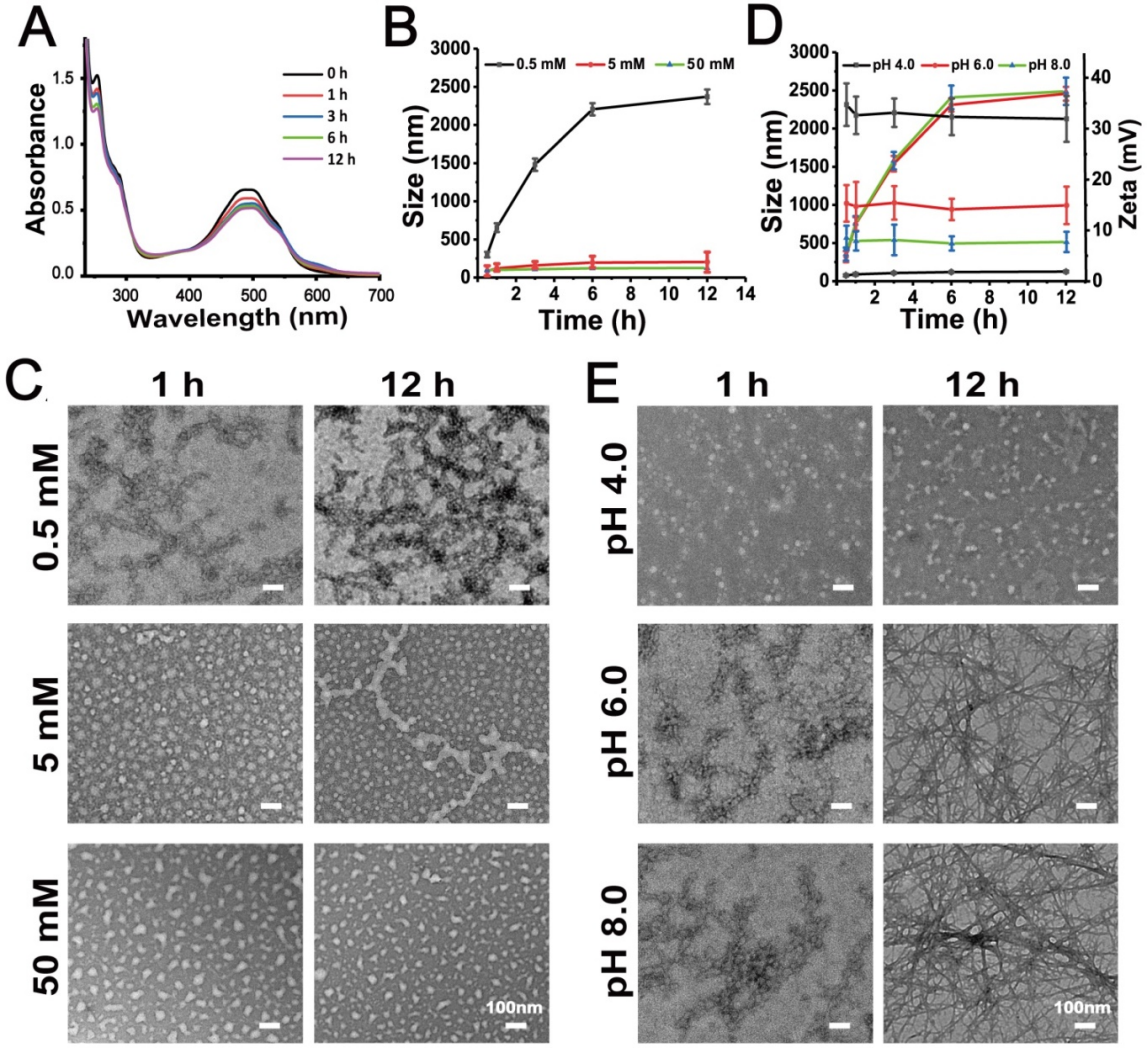
Self-assemble mechanism of DPC-NPs (100 µM). (**A**) UV-vis spectral characteristics of the DPC-NPs self-assembly process. (**B**) Effect of different concentrations of hydrogen bond blocker (urea) on DPCs' size. (**C**) TEM images of DPCs under different concentrations of hydrogen bond blocker (urea). (**D**) Size and Zeta potentials of DPCs at different pH values. (**E**) TEM images of DPCs at different pH values.

Fluorescence spectrophotometry, UV-visible spectrophotometry, DLS and TEM experiments were performed to confirm the self-assembly capacity of DPCs. The critical aggregation concentration (CAC) of DPCs was approximately 20 μM (Figure S10), indicating the good self-assembly capacity of DPCs. DPC is an amphiphilic molecule, and transform into a micelle-like spherical structure (DPC-NPs) driven by hydrophobicity immediately in aqueous solution. In order to investigate the driving forces during self-assembly process of DPC-NPs, including π-π stacking, hydrogen bonding and electrostatic forces, full-wavelength scan of UV-visible spectrophotometry, DLS and TEM were performed 6. Two absorption peaks centered at 265 and 480 nm appeared (Figure 1A), representing the peptide backbone and DOX molecule, respectively, and the decrease in absorption over time revealed the formation of assembled aggregates. In particular, the absorbance at 265 nm decreased in a time-dependent manner, suggesting that DPC molecules aggregated through π-π interactions between aromatic residues 27, 30.

Besides π-π interactions, the effect of hydrogen bonding on assembly behavior was also studied. Increasing concentrations of urea were employed to simulate the increasing intensity of hydrogen bond block effect 31. At the urea concentration of 5 mM and 50 mM, DPC-NPs displayed a constant particle size, suggesting the inhibition of self-assembly process (Figure 1B). Also supporting the conclusion, TEM results showed that most of the DPC-NPs maintained spherical nanoparticle structures when the hydrogen bonding was blocked (urea concertation ≥5 mM), and the nanoparticles could not assemble to crosslinked fibers (Figure 1C). These results indicate that the hydrogen bonding between DPC-NPs contributes to their self-assembly process.

Furthermore, to investigate the effect of electrostatic force on the self-assemble process, we placed the DPCs into PBS with different pH values i.e. 4.0, 6.0, 8.0 32. DPCs exhibited different Zeta potentials at different pH values (Figure 1D). At pH 4.0, Zeta potential was +34.7 mV (1 h), presenting strong electrostatic repulsion, thereby preventing the self-assembly process of DPC-NPs. In contrast, at pH 6.0 and pH 8.0, Zeta potential of DPC-NPs was +15.3 mV and 8.5 mV (1 h), exhibiting weaker electrostatic repulsion compared to that at pH 4.0, and the increase of the particle size revealed the recovery of self-assemble capacity. Consistently, TEM images showed that cross-linked fiber structure could be observed at pH 6.0 and 8.0 (Figure 1E). All the results demonstrated that electrostatic repulsion prevents the DPC-NPs self-assembly.

### The changing Zeta potential of FPG at different pH confirms its acid-responsive capacity

The zeta potential values of FPG at different pH values was measured. In neutral phosphate buffer solution (pH 7.4), FPG exerted a negative potential, while in weakly acidic conditions (pH 6.5), the potential of FPG responsively changed from -30 mV to +35 mV (Figure S11). Distinguished from FPG, ε-PL exhibited a potential higher than +120 mV under both conditions, revealing that the addition of DMMA gave acid-responsive capacity to FPG 29. Once exposed to a mildly acidic environment such as the tumor microenvironment, the amide bond in FPG hydrolyzes, leading to the detachment of DMMA and the removal of the amidogen in ε-PL, which is positively charged under acidic conditions.

### FDPC-NPs properties including Size, Zeta potential, stability and in release profile were evaluated

We first determined the particle size, zeta potential and morphology of prepared FDPC-NPs with DLS and TEM, and the results are shown in Figure S12. The size of FDPC-NPs was 80.85±5.4 nm, and the zeta potential was -9.03±2.85 mV. TEM images showed that the prepared micelles exhibited a uniform spherical shape with a narrow polydispersity. Drug loading content of DPCs in FDPC-NPs is 20.73%, and therefore the amount of FPG in FDPC-NPs is 79.27%.

**Figure 2:**
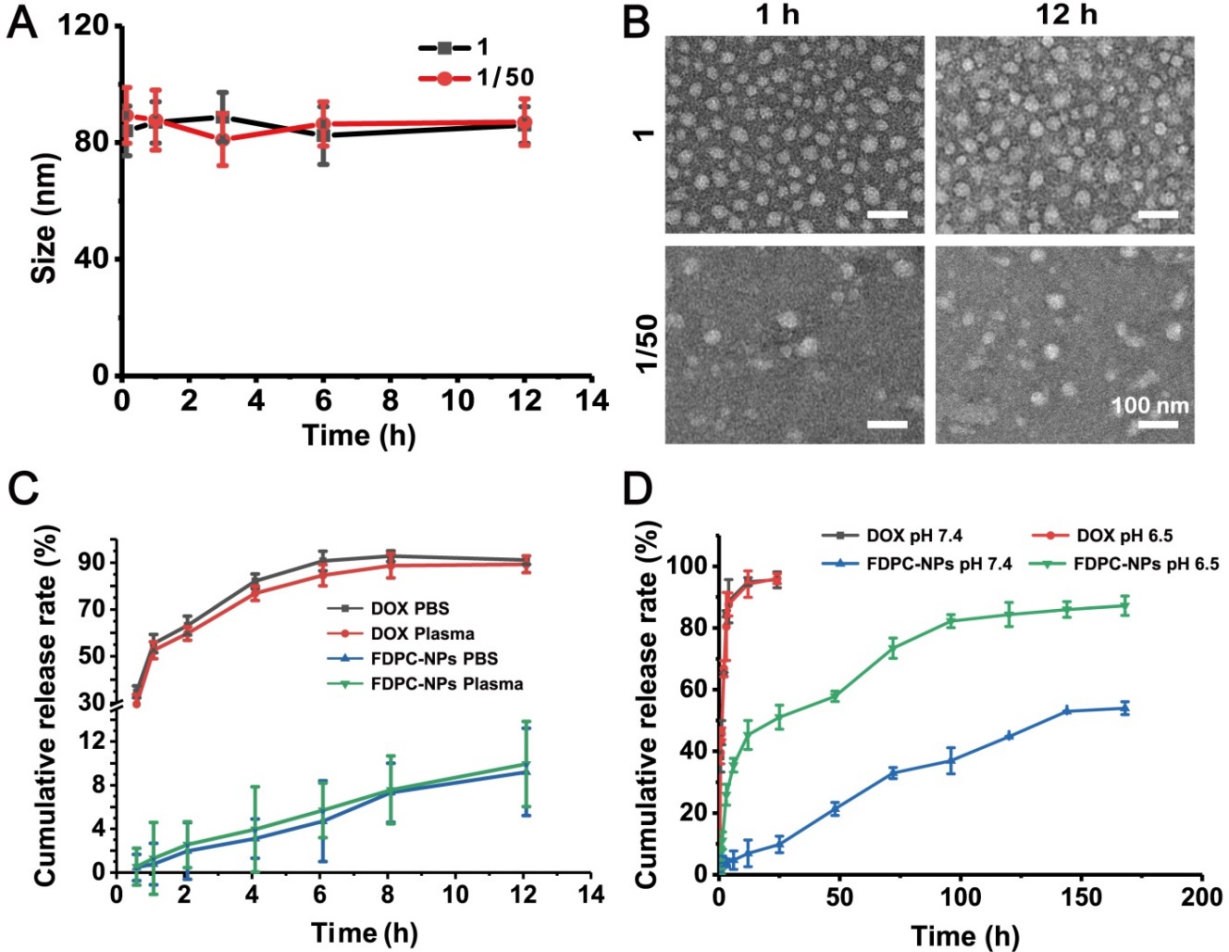
Stability assessment and cumulative release of FDPC-NPs *in vitro.* (**A**) Size change of FDPC-NPs under 50-fold dilution. (**B**) TEM images of FDPC-NPs under 50-fold dilution. (**C**) Release profile of free DOX and FDPC-NPs in PBS and rat plasma. (**D**) Cumulative release of FDPC-NPs in phosphate buffer solution at different pH values.

After injection, FDPC-NPs need to go through blood circulation to reach tumor area and are quickly diluted by blood, therefore, the stability of FDPC-NPs in plasma should be considered. As shown in Figures 2A-B, particle size and morphology of FDPC-NPs did not change after 50-fold dilution, indicating the favorable stability when diluted by blood. Stability of FDPC-NPs in plasma was also evaluated. As shown in Figure 2C, FDPC-NPs in plasma exerted similar releasing behaviors to that in PBS (pH 7.4), indicating the structure of FDPC-NPs would not be destroyed by enzymes and kept stable in the blood.

In our design, when FDPC-NPs reaches an acidic environment, the FPG shell separates from the DPC-NPs core driven by the mutual repulsive effect of the like charges due to the charge reversal of FPG, leading to core exposure and subsequent drug release. An *in vitro* release experiment was carried out to confirm the acid-responsive release behavior of FDPC-NPs. The results showed that FDPC-NPs released more drugs in weakly acidic solution (pH 6.5) than in neutral solution (pH 7.4) (Figure 2D), revealing the good acid-responsive release function of FDPC-NPs. In addition, we can also observe that FDPC-NPs exhibited a sustained release profile compared to free DOX (Figure 2D).

### FDPC-NPs morphology transform from nanoparticles to nanofibers *in vitro*

**Figure 3:**
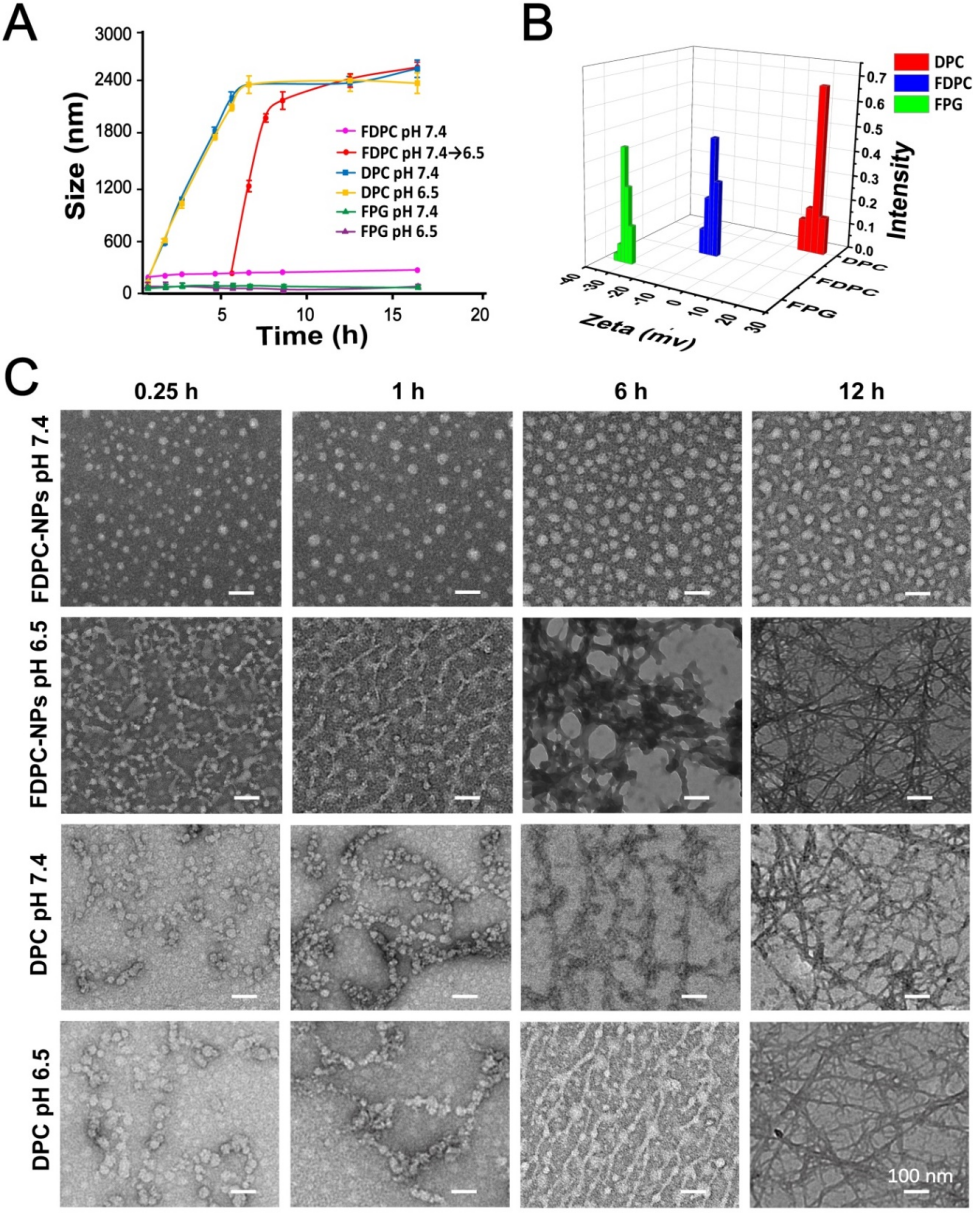
Self-assembly behavior study of FDPC-NPs *in vitro*. (**A**) Acid-responsive sizes of FDPC-NPs, DPCs and FPG at different pH values. (**B**) Zeta potential analysis of FDPC-NPs. (**C**) TEM images of DPCs and FDPC-NPs at pH 6.5 and 7.4 at different time points.

DLS and TEM were performed to monitor the process of the pH change-triggered morphological transformation and zeta potential of the FDPC-NPs (Figures 3A-C). No obvious change in the particle size of the FDPC-NPs was observed in the neutral environment, while the size increased from 80 nm to over 2500 nm when we adjusted the solution pH value to 6.5. In contrast, the particle size of DPCs increased significantly at either pH 7.4 or pH 6.5. These results demonstrate that FPG helps FDPC-NPs maintain their morphology under neutral conditions, and the particle size of FDPC-NPs could responsively increase in weakly acidic environments. Also supporting this conclusion were the TEM images that showed that the FDPC-NPs maintained a uniform spherical size of 80 nm and exhibited good morphological stability in neutral aqueous solution (pH 7.4) but aggregated into a long necklace-like chain structure or a crosslinked fiber structure over time in a weakly acidic solution (pH 6.5). In addition, Figure 3B shows that the FDPC-NPs displayed a zeta potential of approximately -10 mV, which benefits the long-term action of the NPs *in vivo* 10, 17.

In addition, the absorbance changing of FDPC-NPs was used to monitor the kinetics of DPCs transformation (Figure S13). At pH 6.5, the average absorbance of FDPC-NPs decreased in a time-dependent manner due to the morphology transformation and eventually reached to 0.504 by 12h. 43.8% of the FDPC-NPs self-assembled to NFs at the 1st hour and 83.7% transformed into NFs in 3 hours.

**Figure 4:**
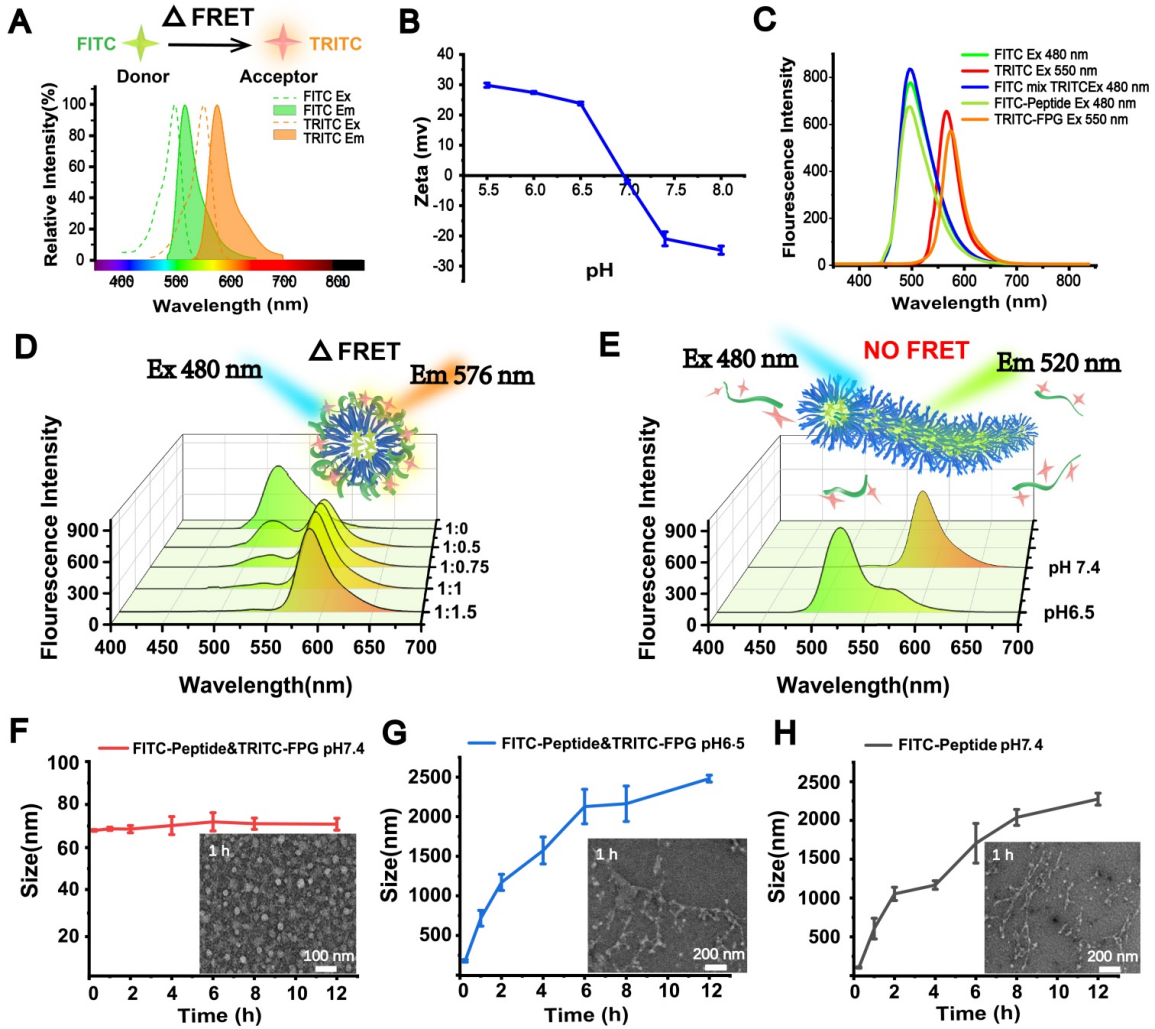
Morphological transformation mechanism demonstrated by FRET *in vitro*. (**A**) Fluorescence spectral overlay of the FRET fluorescence donor (FITC) and receptor (TRITC). (**B**) Zeta potential analysis of TRITC-FPG. (**C**) Fluorescence emission spectra of the FRET fluorescent donor (FITC)-FPG and receptor (TRITC)-labeled peptides. (**D**) Fluorescence emission spectra of FRET-NPs with increased molar ratios of FITC-peptide to TRITC-FPG. (**E**) Fluorescence emission spectra of FRET-NPs at pH 7.4 and pH 6.5. (**F-H**) Size and TEM characterization of FITC-labeled NPs at pH 7.4 (F), FITC-labeled peptide at pH 7.4 (G) and FITC-labeled NPs at pH 6.5 (H).

To further investigate the acid-sensitive transformation mechanism of the FDPC-NPs, we employed fluorescence resonance energy transfer (FRET), an effective method that is widely used to monitor the distance between two molecules 33. In a typical FRET experiment, a donor fluorescein and an acceptor fluorescein are first labeled on the two molecules. When the distance between the two molecules is less than the FRET radius, a FRET signal appears; otherwise, the signal disappears. Here, we utilized fluorescein isothiocyanate (FITC) (λ_ex_=480 nm, λ_em_=520) as the donor and tetramethylrhodamine isothiocyanate (TRITC) (λ_ex_=547 nm, λ_em_=576) as the acceptor to label the self-assembling peptide and FPG, respectively 34, 35. As the preliminary step in FRET, the acid-responsive function of the fluorescently labeled FPG (TRITC-FPG) and the two fluoresceins were examined to ensure the reliability and feasibility of the experiment. Figure 4A shows that the emission spectrum of FITC and the excitation spectrum of TRITC satisfied the overlap requirement of FRET, and Figure 4B displays the charge reversing process of TRITC-FPG, confirming its acid-responsive function.

Prior to FRET detection, the fluorescence emission spectra of the donor (FITC-peptide) and acceptor (TRITC-FPG) were scanned separately, revealing that the fluorescence spectra of the donor and acceptor were not altered after modification. In addition, separate and mixed free-state fluorescent solutions (20 µM each FITC and TRITC) were also detected, and no FRET signal was observed (Figure 4C). Next, FITC-peptide and TRITC-FPG were co-incubated in a neutral environment (pH 7.4) with an increasing molar ratio from 1:0.5 to 1:1.5. Along with the increasing ratio, the FITC-peptide signal disappeared, while significant FRET signals were observed (Figure 4D), indicating that FITC-peptide and TRITC-FPG were close in proximity to each other and probably formed spherical FRET-nanoparticles (FRET-NPs) in solution. According to the result that the strongest FRET signal appeared at the molar ratio of 1:1 and no obvious promotion occurred at the ratio of 1:1.5, we chose 1:1 as our subsequent experimental ratio. Furthermore, when we changed the pH value of the solution from 7.4 to 6.5, the FRET signal disappeared, and the FITC signal reappeared (Figure 4E), suggesting that the TRITC-labeled FPG and the FITC-labeled peptide separated from each other and that the distance between them exceeded the FRET radius.

Moreover, the particle size and morphology of the FRET-NPs in solutions with different pH values were also determined with DLS and TEM. The FRET-NPs maintained their nanoparticle morphology at pH 7.4 (Figure 4F) and transformed into fibers with increasing particle size at pH 6.5 (Figure 4G). Moreover, Figure 4H shows that the FITC-peptide self-assembled into fibers, and the particle size increased at pH 7.4. These results demonstrate that the FITC-labeled peptide exerted a similar assembly behavior to that of DPCs.

### FRET confirms the core-shell separation process* in vivo*

**Figure 5:**
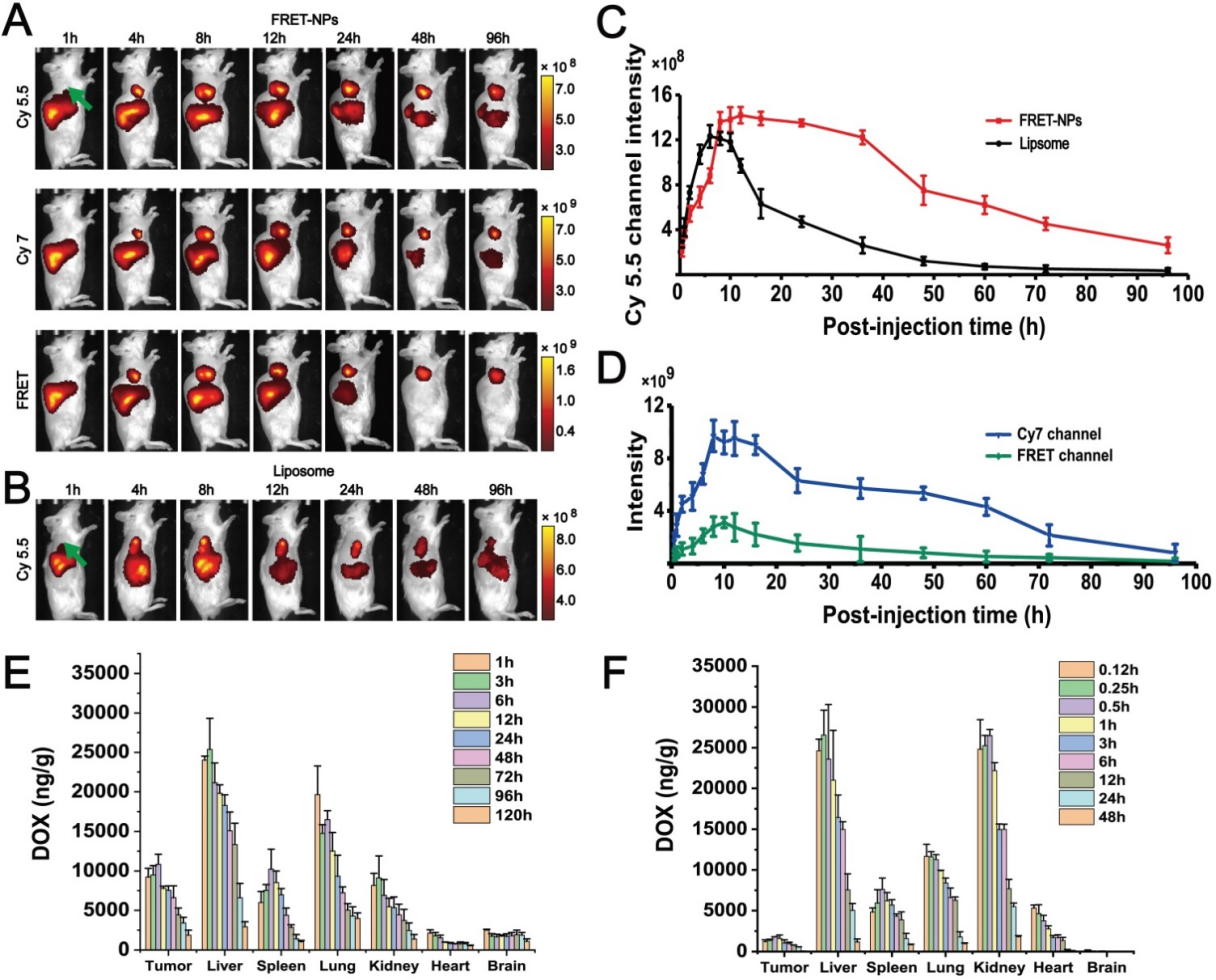
Distribution behavior study of FDPC-NPs *in vivo*. (**A**) Representative NIRF images obtained at selected postinjection times in three optical channels: Cy5.5 (λ_ex_=640 nm and λ_em_=720 nm); Cy7 (λ_ex_=745 nm and λ_em_=800 nm); and FRET (λ_ex_=640 nm and λ_em_=800 nm) with (**B**) Cy5.5 (λ_ex_=640 nm and λ_em_=720 nm)-labeled liposomes as a control. The right flank tumors are marked with green arrows. (**C**) The fluorescence intensities of the FDPC inner core and liposome (control) in the tumor area. (**D**) The fluorescence intensities of the FDPC outer shell and FDPC in the tumor area. (**E-F**) Biodistribution of DOX accumulation in different organs for the FDPC-NPs group. (E) and DOX solution group (F) (n =6).

FRET imaging was also conducted* in vivo*. Cy5.5 (λ_ex_=640 nm, λ_em_=720) and Cy7 (λ_ex_=745 nm, λ_em_=800) were selected as the donor and acceptor to form a FRET pair^27^. Two groups of H22 hepatocarcinoma tumor-bearing mice were intravenously injected with FRET-NPs and Cy5.5-labeled liposomes (with a similar particle size to FRET-NPs but could not self-assemble) as a control. Both of them had a particle size of approximately 80 nm (Figure S14). We applied an imaging system for small animals *in vivo* to record images in the channels of Cy5.5, Cy7 and FRET at several time points from 1 to 96 h (Figures 5A-B). For the FRET-NPs group, the FRET, Cy5.5, and Cy7 signal intensities represented the content of FRET-NPs, the inner core and the outer shell, respectively. The distribution of liposomes *in vivo* was characterized by detecting the Cy5.5 signal. The FRET signal in Figure 5A revealed that the FRET-NPs maintained a stable structure during the transport process in blood vessels. The average intensities of the different channels belonging to the tumor areas are summarized in Figures 5C-D. The significant increasing signal intensities of all channels after intravenous injection revealed the enrichment of FRET-NPs and liposomes at the tumor site where they gradually decreased after 8 h, indicating that the particles were eliminated by circulation. Notably, the decrease rate of liposomes was higher than that of the inner core in FRET-NPs (Figure 5C), indicating the long retention effect of the FRET-NPs. In addition, the rate of decrease of Cy7 and FRET signal intensities was higher than that of Cy 5.5 in the group of FRET-NPs after 8 h (Figures 5C-D), showing that the FRET-NPs separated into the outer shell and the inner core, and that the outer shell was cleared quickly by blood circulation. These results indicate that the inner core of the FRET-NPs self-assembled into fibers to achieve a long retention effect.

### FDPC-NPs exerts long-term retention effect in tumor-bearing mice

To further confirm the long retention effect of FDPC-NPs *in vivo*, the biodistribution of FDPC-NPs was determined in H22 hepatocarcinoma-bearing mice with UPLC-MS/MS. The drug concentration in the tumors of the FDPC-NPs group was markedly higher than that of the DOX solution group at all time points (Figures 5E-F), and the relative tumor uptake rate of the FDPC-NPs group was 31 times higher than that of the DOX solution group. In addition, the drug concentration in other tissues, especially the heart and kidney, of the FDPC-NPs group was less than that of the DOX solution group (Figures 5E-F), which might contribute to reduced cardiorenal cytotoxicity and enhanced safety. Furthermore, the half-life of DOX in the tumors of the FDPC-NPs group was 5.2 times higher than that of the DOX solution group, revealing the long-term retention effect of FDPC-NPs.

### FDPC-NPs displayed cytotoxicity against tumor cells

**Figure 6:**
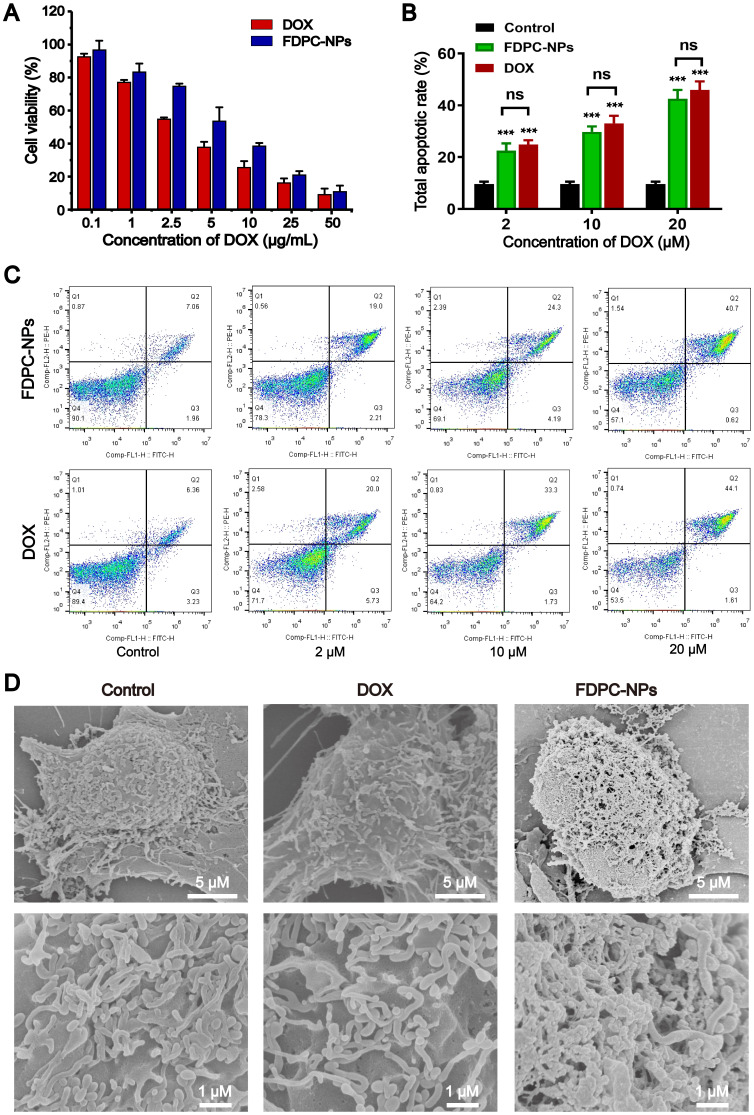
Pharmacological activity evaluation *in vitro*. (**A**) Relative viability of SMMC-7721 treated with different concentrations of FDPC-NPs and DOX for 24 h. (**B-C**) Apoptosis rates of SMMC-7721 cells incubated with FDPC-NPs and DOX. *P < 0.05 and **P < 0.01 compared with saline control. (**D**) SEM images of SMMC7721 cells treated with 200 µl of DMEM, DOX (50 µM) or FDPC-NPs (50 µM) for 12h.

We treated SMMC-7721 cells with different concentrations of FDPC-NPs, DOX, peptide and FPG, and cell viability was detected with the MTT assay. As shown in Figure S15, both the peptide and FPG exhibited no obvious cytotoxicity, while FDPC-NPs and DOX displayed cytotoxicity against tumor cells (IC_50 DOX_ = 2.965 μg/mL; IC_50 FDPC-NPs_ = 5.896 μg/mL) (Figure 6A). Additionally, the results from flow cytometry with annexin-V-FITC/PI double staining showed that FDPC-NPs and DOX significantly increased the proportion of apoptotic cells in a concentration-dependent manner, and the two groups exhibited similar cytotoxicity (Figures 6B-C). After pH-trigged shell-core separation, FDPC nanoparticles transforms into nanofibers in cell culture medium (Figure 6D), and the fibers cannot be uptake directly by tumor cells. DOX gradually releases along with the hydrolysis of peptides in fibers, and only free DOX which is dissociated from DPCs can be uptake (Figure S16). Therefore, FDPC-NPs tumor cell inhibitory efficiency is a little weaker than that of free DOX in 24 h. In addition, we also treated normal mammalian liver cells (LO2 cell) with different concentrations of peptide and FPG. As shown in Figure S15, the peptide and FPG displayed no obvious cytotoxicity against LO2 cells, revealing the certain safety of FDPC-NPs.

The self-assembly process of FDPC-NPs in cell culture medium was monitored with SEM. As shown in Figure 6D, cells treated with PBS or DOX exhibited numerous irregular protrusions on the surface *i.e.* microvillus and pseudopodia, while for FDPC-NPs treated cells, serried fiber structures were clearly observed and the fibers covered the surface of the tumor cells, attributing to the self-assembly of DPCs, which was in accordance with the TEM images in FDPC-NPs morphology observation (Figure 3C). Due to the negatively charged phosphatidylserine in the cell membrane, positively charged DPCs adhered on the surface of SMMC7721 cells. With the increasing incubation time of FDPC-NPs with SMMC7721 cells, the pH of culture medium changed gradually from neutral to weakly acid (pH 6.5-6.8), allowing the DPC cores separating from the FPG shells and self-assembling into DPC fibers. These results reveals FPDC-NPs self-assembles into fibers and adheres on the surface of the tumor cells in cell culture medium, confirming that FPDC transform from NPs to NFs in tumor weakly acid environment.

### FDPC-NPs exerts long-term antitumor effect on tumor-bearing mouse model

**Figure 7:**
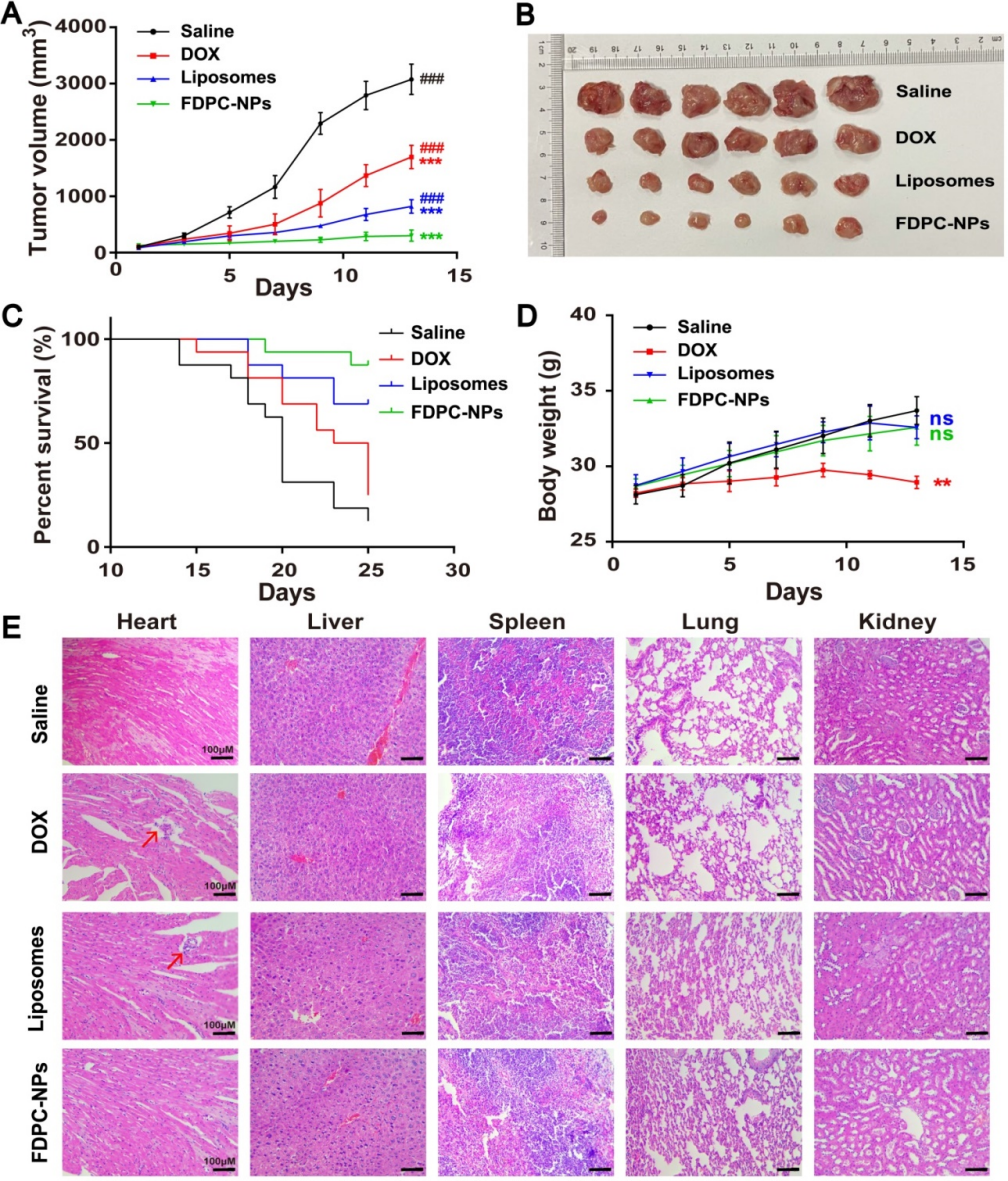
*In vivo* antitumor effects of saline, DOX solutions, DOX liposomes and FDPC-NPs on H22 tumor-bearing mice. (**A**) Tumor volume variation profiles. ***P < 0.001 compared with saline and ^###^ P< 0.001 compared with FDPC-NPs. (n=6) (**B**) Photograph of tumor tissues excised from H22 tumor-bearing mice on day 13. (**C**) Cumulative survival rate of H22 tumor-bearing mice (n=15). (**D**) Body weight changes of H22 tumor-bearing mice. **P < 0.01, ns P > 0.05 compared with saline. (**E**) Representative microphotographs of H&E-stained organic tissues excised from H22 tumor-bearing mice on day 13 (scale bar =100 µm).

An H22 hepatocarcinoma tumor-bearing mouse model was employed to test the pharmaceutical effect of FDPC-NPs. When the tumor volume reached approximately 100 mm^3^, the mice were randomly divided into four groups, and treated with (1) saline; (2) DOX solution; (3) DOX-liposomes or (4) FDPC-NPs respectively. DOX-liposomes exhibit similar size to FDPC-NPs, but do not possess FDPC-NPs' pH-triggered morphology transformation capacity. The tumor sizes and body weights were measured and recorded each other day. Considering the long retention effect of the FDPC-NPs, the frequency of administration was set to once every three days. At the end of the experiment (day 13), the mice were sacrificed and dissected, and the tumors were photographed. Results showed that DOX solution, DOX-liposomes and FDPC-NPs displayed significant therapeutic effects against tumors (P < 0.001). Particularly, DOX-liposomes and FDPC-NPs behaved better (Figures 7A-B) due to the EPR effect. Nano-sized liposomes and FDPC-NPs could extravasate through the tumor vasculature, and enter into cytoplasm through cellular uptake. Because of the pH-triggered morphology transformation capacity, FDPC-NPs transformed from nanoparticle to nanofiber in weakly acid tumor tissues, thus avoid being eliminated by circulation, resulting in a favorable inhibition effect (P < 0.001) compared with DOX-liposomes. Moreover, based on the survivorship curves, treatment with FDPC-NPs remarkably promoted the survival rate of tumor-bearing mice, which furtherly confirmed the therapeutic effect and biological safety of FDPC-NPs (Figure 7C).

Body weights variation and hematoxylin and eosin (H&E) staining confirmed the safety of DOX-liposomes and FDPC-NPs. As shown in Figure 7D, a significant gradual decline of the body weights in DOX group was observed compared to the saline group (P < 0.01), indicating the potential toxicity of DOX solution. On the contrary, the administration of DOX-liposomes and FDPC-NPs barely influenced the body weights of the model mice, revealing the safety of DOX-liposomes and FDPC-NPs. Consistently, the organs (heart, liver, spleen, lung and kidney) of mice in four groups were collected for H&E staining. DOX exhibited significant heart toxicity while FDPC-NPs did not, and no obvious pathological damage or inflammatory lesion was observed in other organs from FDPC-NPs-treated mice, which further indicated the biosafety of FDPC-NPs. In addition, after treating with FPDC-NPs, the concentration of IL-1β, IL-6 and TNF-α in mice sera did not elevate relative to the saline control (P > 0.05), demonstrating the low immunogenicity of FPDC-NPs (Figure S17).

## Conclusion

In this study, we introduced novel transformable morphology FDPC-NPs to prolong drug retention time in tumor lesions based on self-assembly peptides. FDPC-NPs are stable during blood circulation and are able to target tumor issues because of the EPR effect, which contributes to the reduced distribution in normal tissues. Once the FDPC-NPs reach the tumor site, the acid-responsive FPG spontaneously separates from the DPC-NPs in the mildly acidic tumor microenvironment, and DPC-NPs subsequently assemble into DPC-NFs driven by π-π stacking, leading to long-term drug retention in tumors. The process of shell-core separation and self-assembly was verified by TEM, SEM and FRET, and the antitumor effects of the FDPC-NPs were confirmed *in vivo* and *in vitro*. In summary, morphology-transformable FDPC-NPs represent a promising therapeutic approach for prolonging the residence time of drugs at the tumor site to reduce heart toxicity and enhance therapeutic efficacy.

## Supplementary Material

Supplementary methods and figures.

## Abbreviations

DOX: doxorubicin
FDPC-NPs: functional doxorubicin-peptide conjugate nanoparticles
DPCs: doxorubicin-peptide conjugates
FPG: functional polylysine graft
DPC-NPs: DPC nanoparticles
DPC-NFs: DPC-nanofibers
EPR: effect enhanced permeability and retention
FRET: fluorescence resonance energy transfer
RP-HPLC: reversed phase high performance liquid chromatography
UPLC: ultra-performance liquid chromatography
MALDI-TOF-MS: matrix-assisted laser desorption/ionization time-of-flight mass spectrometry
SPPS: solid-phase peptide synthesis
CAC: critical aggregation concentration
FITC: fluorescein isothiocyanate
TRITC: tetramethylrhodamine isothiocyanate
FRET-NPs: FRET-nanoparticles
