# Supplementary material for: Morphological transformation enhances Tumor Retention by Regulating the Self-assembly of Doxorubicin-peptide Conjugates
Source: Theranostics. 2020 Jul 9;10(18):8162–78. doi: 10.7150/thno.45088 (PMC7381745; doi:10.7150/thno.45088)
Supplement: Supplementary file 1 — Supplementary methods and figures. [file thnov10p8162s1.pdf]

Supporting Information

Morphological transformation enhances tumor retention by regulating the self-assembly of doxorubicin-peptide conjugates

Liu Xu[a,‡], Yutong Wang[a,‡], Chenqi Zhu[a,b ‡], Shujing Ren[a], Yurou Shao[a], Li Wu[a], Weidong Li[a], Xiaobin Jia[c], Rongfeng Hu[d], Rui Chen[a,*], Zhipeng Chen[a,*]

[a] College of pharmacy, Nanjing University of Chinese Medicine, Nanjing 210023, China

[b] Department of Pharmacy, the Affiliated Suzhou Hospital of Nanjing Medical University, Suzhou 215002, China

[c] School of Traditional Chinese Pharmacy, China Pharmaceutical University, Nanjing 211198, China

[d] Key Laboratory of Xin'an Medicine, Ministry of Education, Anhui University of Chinese Medicine, Hefei, Anhui 230038, People's Republic of China.

‡These authors contributed equally.

Corresponding Author

*E-mail addresses: czpcpu@sina.com (Z Chen). carol-chen-07@126.com (R. Chen).

Chemicals and general methods

Chemicals and materials: Fmoc-amino acids and Rink Amide resin (100-200 mesh, substitution factor: 0.486 mM) were obtained from GL Biochem (Shanghai, China). O-Benzotriazole-N,N,N',N'-tetramethyl-uronium-hexafluorophosphate (HBTU, >98%), N,N-diisopropylethylamine (DIEA, >98%), (3H-1,2,3-triazolo[4,5-b]pyridin-3-yloxy)tri-1-pyrrolidinium hexafluorophosphate (PyAOP, >98%) and doxorubicin hydrochloride (DOX, >95%) were obtained from Aladdin (Shanghai, China). Piperidine was obtained from Nanjing University of Chinese Medicine. Triisopropylsilane (TIS, >98%) and trifluoroacetic acid (TFA, 99%) were obtained from Macklin (Shanghai, China). N,N-Dimethyl-formamide (DMF, 99%) and triethylamine were purchased from Nanjing Chemical Reagent Co., Ltd. Hydrochloric acid (36–38%, HCl) was purchased from Shanghai Ling Feng Chemical Reagent Co., Ltd. (Shanghai, China). Sodium hydroxide (≥96%, NaOH) was purchased from Nanjing Chemical Reagent Co., Ltd. (Nanjing, China). Formic acid was obtained from Tianjin Damao Chemical Reagent Factory (Tianjin, China). Dimethyl sulfoxide (DMSO, 99.9%) was purchased from Sigma (St. Louis, MO, USA). Uranyl acetate (>99.9%) and 2,3-dimethyl maleic anhydride (DMMA, >99%) were obtained from Xi'an Dingtian Chemical Co. Ltd. (Xi'an, China). 3-(4,5-Dimethylthiazol-yl)-2,5-diphenyltetrazolium bromide (MTT) was purchased from Sigma (St. Louis, MO, USA); phosphate-buffered saline (PBS, Gibco®) was purchased from Thermo Fisher Scientific (USA); and LysoTracker® Red DND-99, MitoTracker Red (FPGS), trypsin, fetal calf serum and DMEM (Gibco®) were purchased from Thermo Fisher Scientific (Waltham, USA). Methanol and acetonitrile were purchased from TEDIA Company, Inc. (USA). Deionized distilled purified water was used for the preparation of all solutions, and the other reagents were HPLC grade and used as received from commercial sources. The ICR mice were purchased from the Experimental Animal Center at Nanjing University of Chinese Medicine, China.

General methods: HPLC was conducted using a Shimadzu LC-20A HPLC (Japan) system with a C18 RP column with acetonitrile and purified water (0.1% TFA) as the eluents. The purification was performed on a preparative RP-HPLC with a global chromatography column (5 μm, 20 mm ID×250 mm) at 25°C on a Varian ProStar Model 325 preparative HPLC (Agilent Technologies, Santa Clara, CA) equipped with a fraction collector. A purified water/acetonitrile gradient of 27-40% was run for 25 min containing 0.1% v/v TFA and used as the eluent at a flow rate of 20 mL/min for peptides and derivatives (DPC or different fluorescently labeled peptides). The identity and purity of all synthesized materials were confirmed using LC-MS analysis. The LC-MS system was composed of an LC-20AB pump, SPD-M20A diode array detector, SIL 20AC autosampler, CTO-20A column oven (Shimadzu, Japan) and 4000 QTRAP® mass spectrometer (AB SCIEX, USA) equipped with a Turbo ion source. Analyst software (Version 1.5.1) and Multi Quant software (Version 1.5.1) were used for data acquisition and analysis, respectively. An Agilent Technologies RP-18 (250 mm × 4.6 mm, 5 μm, Agilent Technologies, Japan) was employed for the separation of analytes at a flow rate of 1 mL/min. The column temperature was 25 °C, and the wavelength was set at 480 nm. The mobile phase consisted of acetonitrile and 0.1% TFA, and a gradient method was employed for the analysis. DLS was performed on a Malvern zeta potential analyzer (Zetasizer Nano ZS90). Fluorescence spectra were recorded on a PerkinElmer LS 55 fluorescence spectrophotometer. TEM images were obtained on a Hitachi transmission electron microscope operating at 120 kV. A BioMate 3S UV/Vis spectrophotometer was utilized for UV-vis full-spectrum scanning (Thermo Fisher Scientific, USA). A FreeZone 4.5 freeze dryer (Labconco, USA) was used for lyophilization to obtain pure product. MTT detection was performed at 570 nm using a microplate spectrophotometer (SpectroAmaxTM 250). The apoptosis rate was analyzed using a BD FACSCalibur flow cytometer (USA). NIRF imaging was performed using a PE IVIS Spectrum imaging system (Lumina LT).

Synthesis of peptide (KIGLFRWR)

The peptide was prepared by solid-phase peptide synthesis (SPPS) using Rink Amide resin and the corresponding N-Fmoc-protected amino acids with side chains properly protected by a Fmoc group or Pbf group. Amino acid couplings were carried out using HBTU and DIEA (resin/amino acid/HBTU/DIEA 1:5:5:10). Piperidine (20%) in anhydrous DMF was used during deprotection of the Fmoc group. Then, the next Fmoc-protected amino acid was coupled to the free amino group using HBTU mixed with DIEA as the coupling reagent. The growth of the peptide chain was performed according to the established Fmoc SPPS protocol. After the last coupling step, excessive reagents were removed by a single DMF wash 5 times (10 mL per gram of resin), 3 min per wash. The product was cleaved using 95% TFA with 2.5% TIS and 2.5% H_2O for 60 min. Then, 30 mL per gram of resin of ice-cold diethyl ether was added to the cleavage reagent. The resulting precipitate was centrifuged for 10 min at 4 °C at 10,000 rpm. Afterward, the supernatant was decanted, and the resulting solid was dissolved in methanol for HPLC separation using acetonitrile and H_2O containing 0.1% TFA as eluents. MS was used to confirm the product.

CAC determination of DPCs

Pyrene methanol solution with a concentration of 1×10^{-4} M was prepared. A total of 50 μL of the pyrene methanol solution was separately added to five centrifuge tubes. Then, the methanol was naturally evaporated and dried in the dark. After that, 5 mL of different concentrations (10, 20, 50, 75, 100 and 200 μM) of DPC aqueous solution was added to each centrifuge tube, and the tubes were sonicated for 5 min to fully disperse the pyrene in the DPC solution. After standing in the dark for 6 h, fluorescence spectrophotometry was performed to measure the emission spectrum of pyrene. The excitation wavelength was set at 334 nm, the excitation slit was set to 5.0 nm, the emission slit was set to 1.5 nm, the scanning speed was medium, and the scanning range was 300 to 600 nm. The ratio of the peak intensity

of the first vibration region to the peak intensity of the third vibration region (I1/I3) was plotted against the logarithm of the concentration of the DPC solution based on the fluorescence spectrum of pyrene. The CAC is defined as the abscissa value of the intersection of horizontal tangent of the curve and the tangent of the curve inflection point .

Cell uptake

SMMC-7721 cells were inoculated in 48-well plates (2×10^4 cells/well). The FDPC-NPs were added to the cells at a concentration of 25 μM of DOX equivalent. After incubating for 12 h, the cells were washed with PBS and the average fluorescence intensity of SMMC-7721 cells were measured by a fluorescence microscopy.

Cytokine Profiling

Healthy ICR mice were randomly divided into five groups (n=6) and treated via the tail vein with saline control, FPG, DOX solution, DOX liposomes or FDPC-NPs at 10 mg DOX equivalent per kg. Collect about 0.4 mL of blood from mice 24 h after injection, and centrifuge to collect serum. Cytokines' (IL-1β, IL-6 and TNF -α) concentrations in serum were determined using enzyme linked immunosorbent assay (ELISA).

Preparation of DOX liposomes

Blank liposomes were prepared by the ethanol injection method followed by ultrasound. Briefly, 120 mg of phosphatidylcholine and 10 mg of cholesterol (6:1 molar ratio) were dissolved in the anhydrous alcohol and then dripped uniformly into 4 mL of ammonium sulfate solution (200 mM) at 25 °C, followed by removing organic solvent via rotary vacuum evaporation. The crude liposomes were then sonicated at 200 W for 40 times to obtain homogenous size distributions and dialyzed for 2 h to remove the redundant ammonium sulfate. An ammonium sulfate gradient method was used for drug loading. 4 mL of doxorubicin hydrochloride (2 mg/mL) was added to the prepared 4 mL of blank liposomes

solutions. Subsequently, samples were stirred for 10 min at 25 °C followed by dialysis for 5 h to remove redundant free DOX.

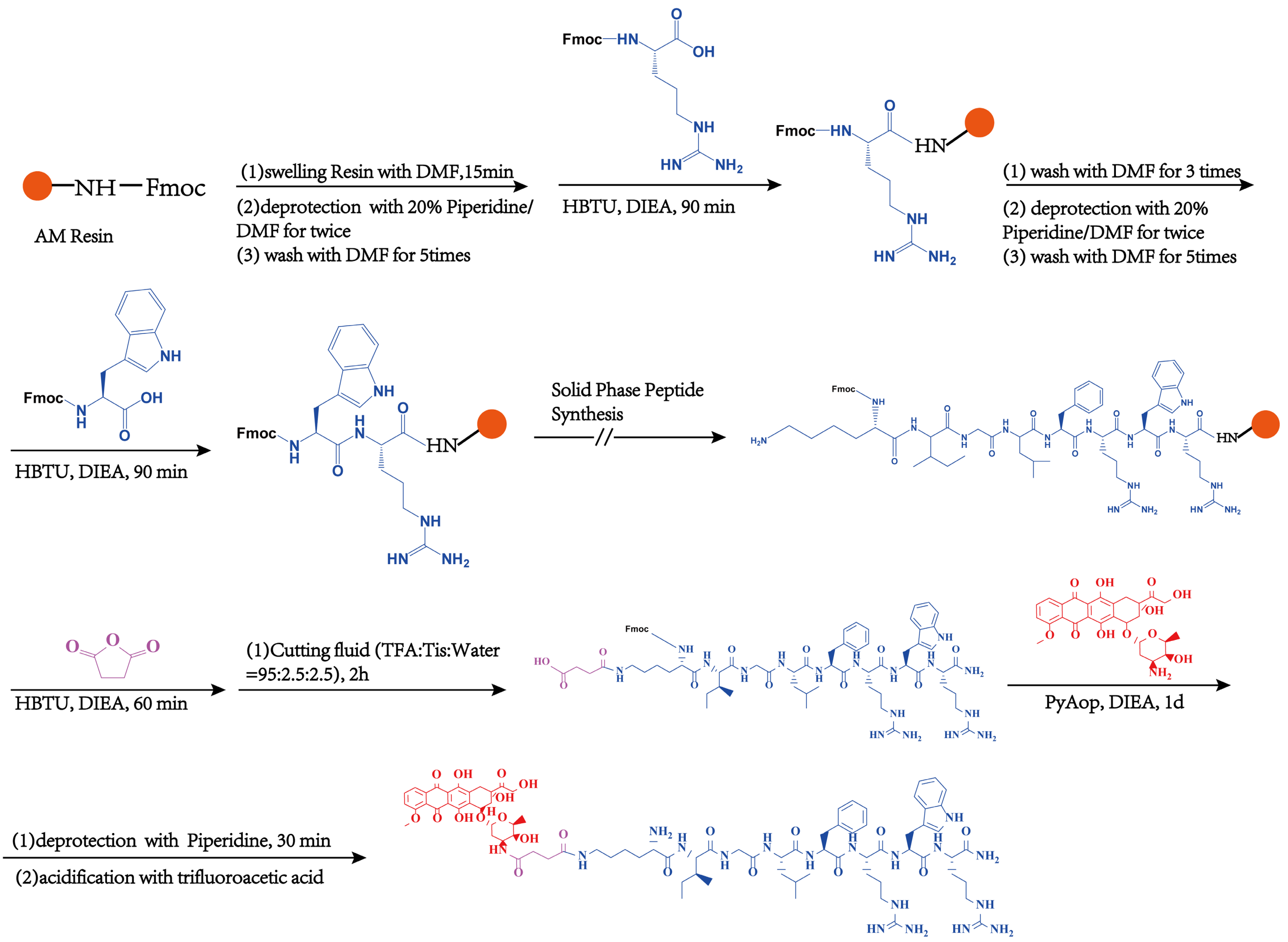

Figure S1. The synthetic route of DPC.

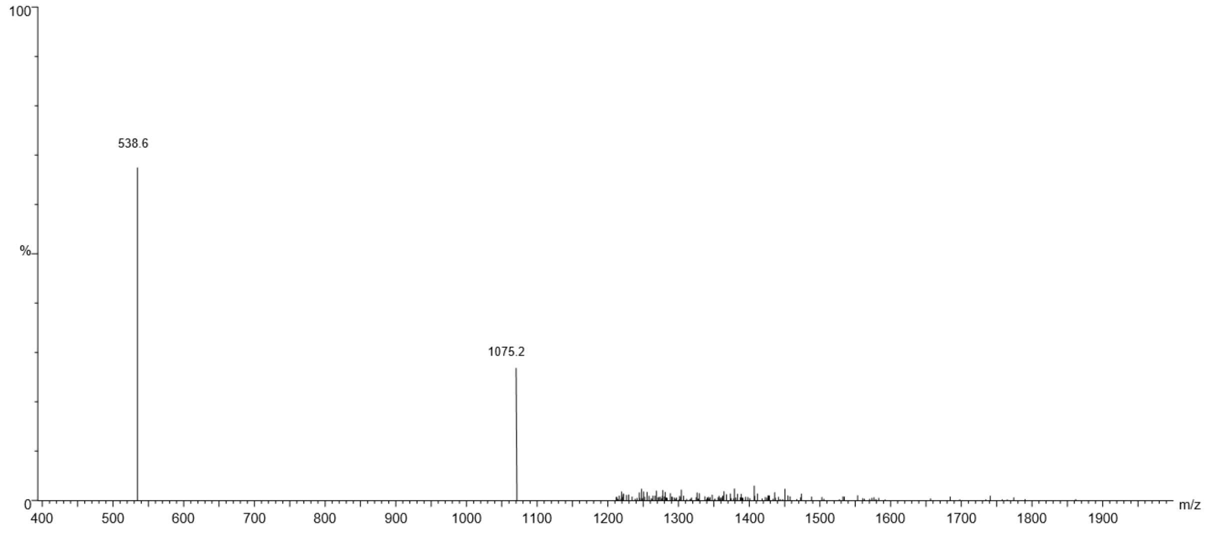

Figure S2. MS spectrum of KIGLFRWR.

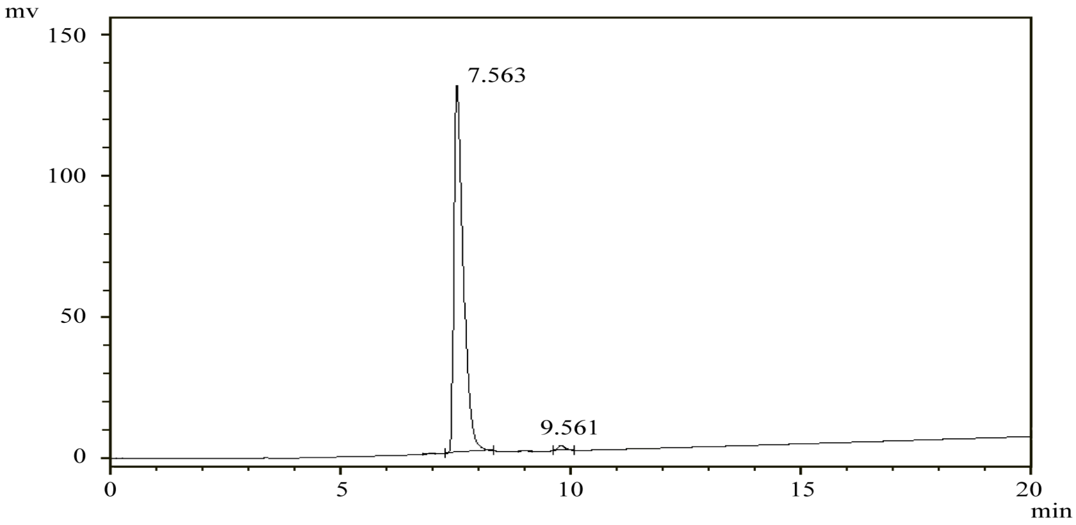

Figure S3. HPLC chromatography of KIGLFRWR.

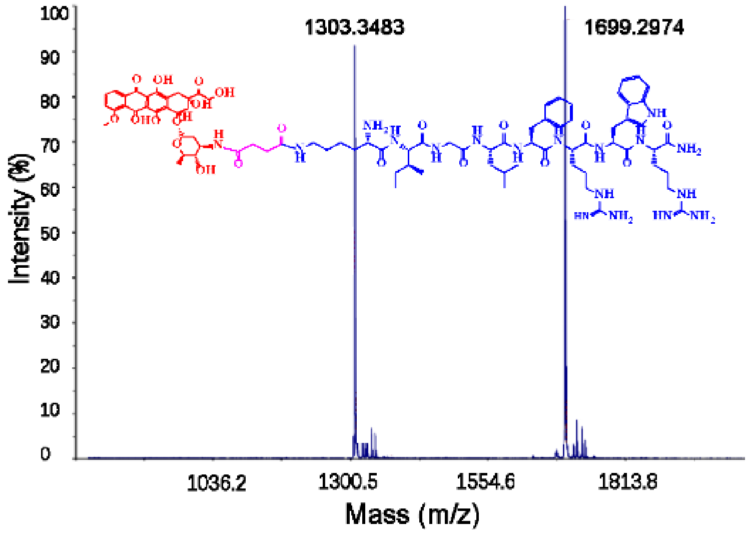

Figure S4. MALDI-TOF MS spectrum of DPC.

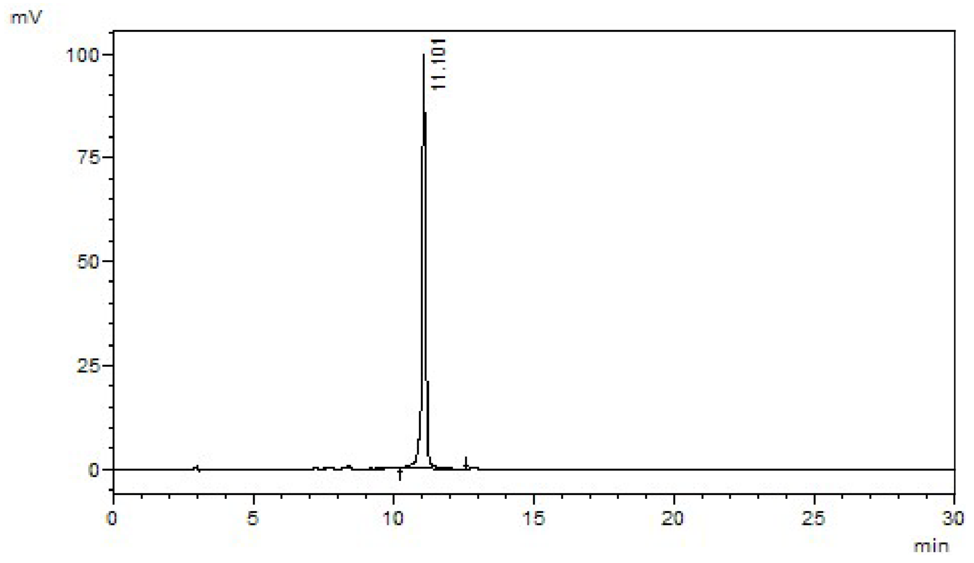

Figure S5. HPLC chromatography of DPC.

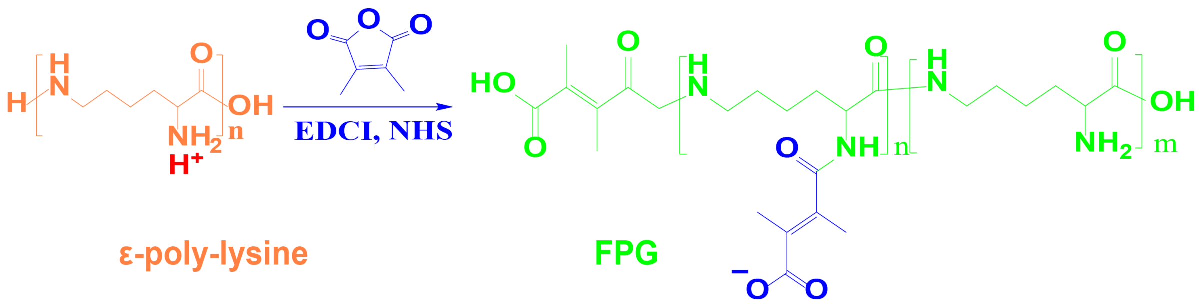

Figure S6. The synthetic route of FPG.

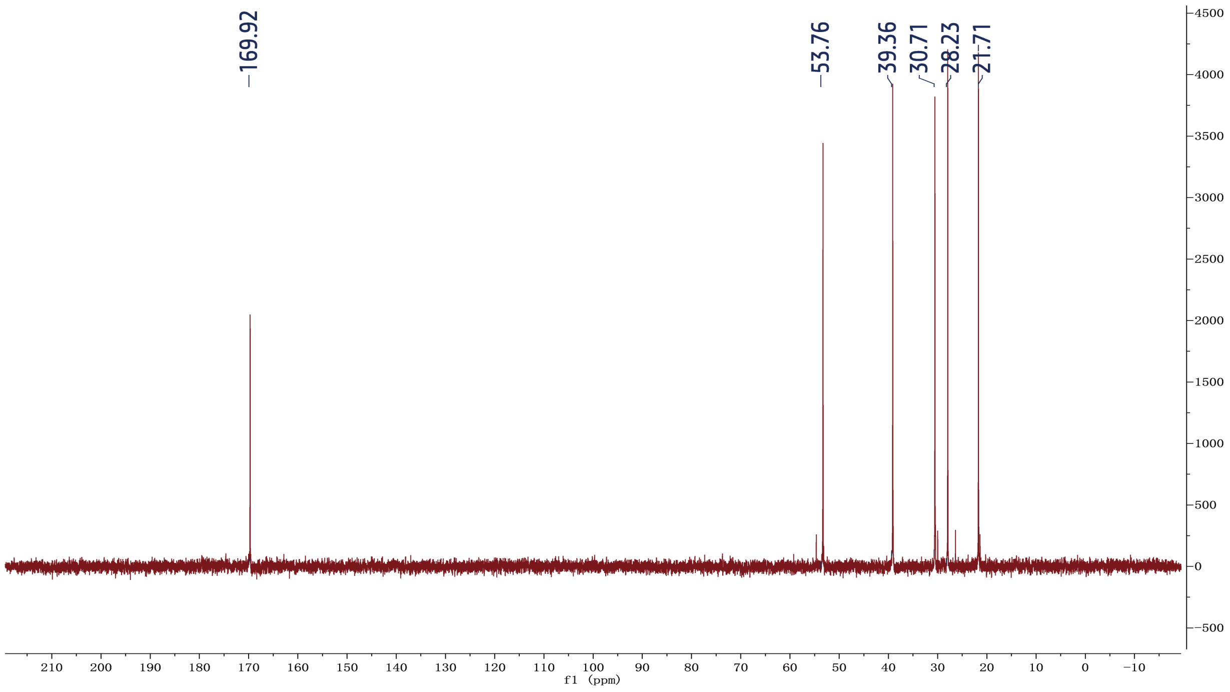

Figure S7. C-NMR spectrum of ε-PL.

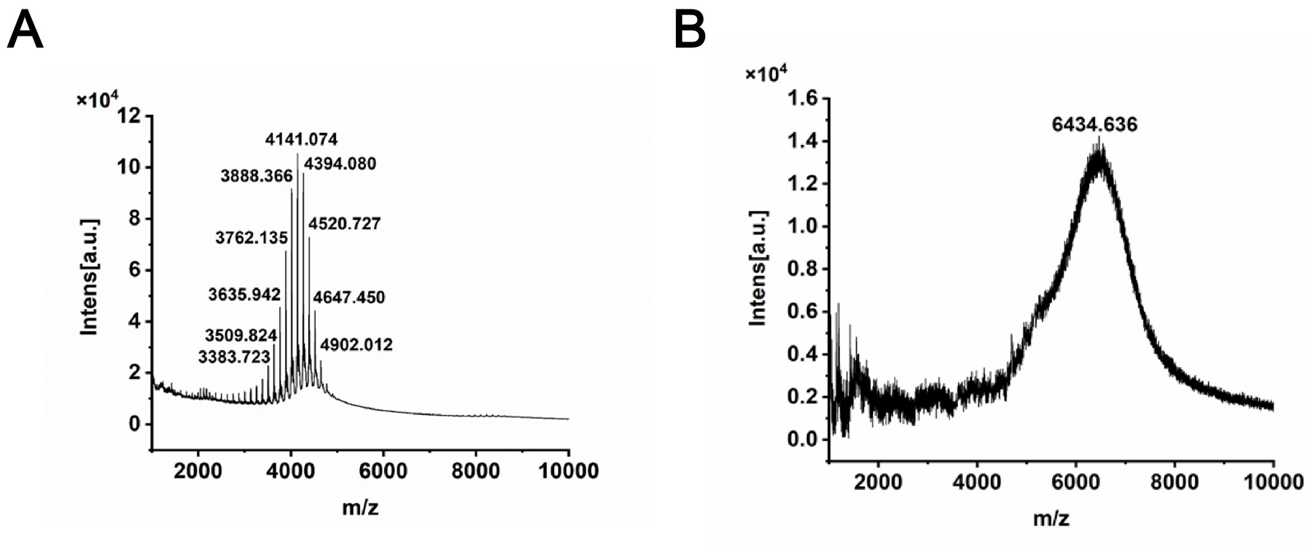

Figure S8. (A) MALDI-TOF MS spectrum of ε-PL. (B) MALDI-TOF MS spectrum of FPG.

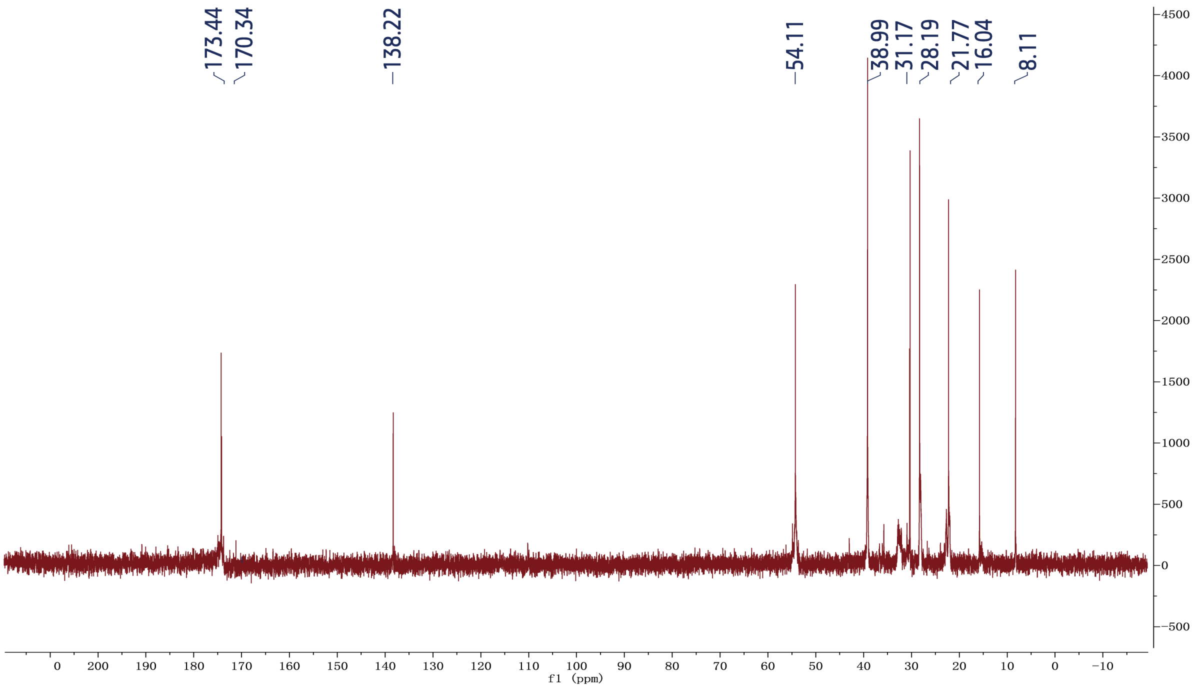

Figure S9. C-NMR spectrum of FPG.

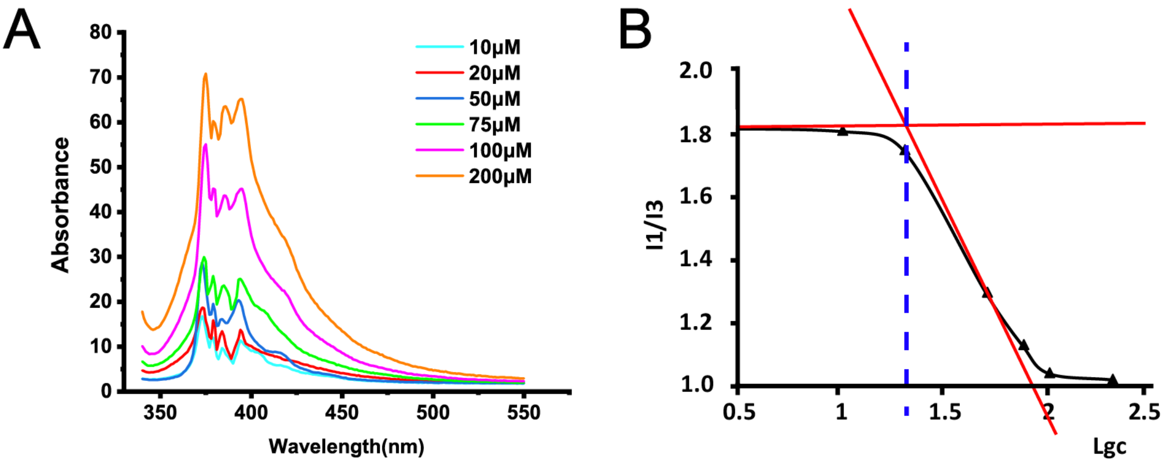

Figure S10. CAC determination of DPCs. (A) Fluorescent emission spectra of pyrene with the increased concentration of DPC and (B) Fluorescence intensity ratio (I_{373nm}/I_{384nm}) of pyrene versus the DPC concentration.

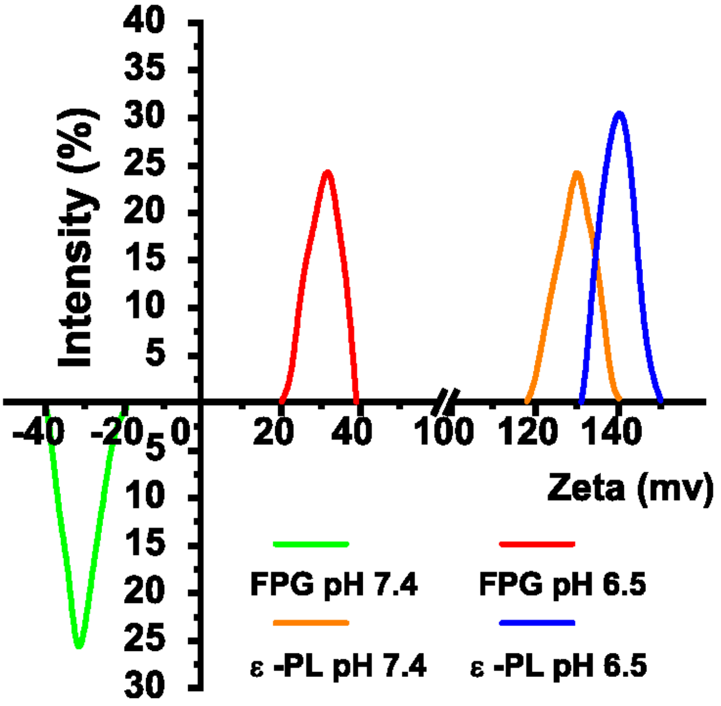

Figure S11. Zeta potential of FPG and ε–PL.

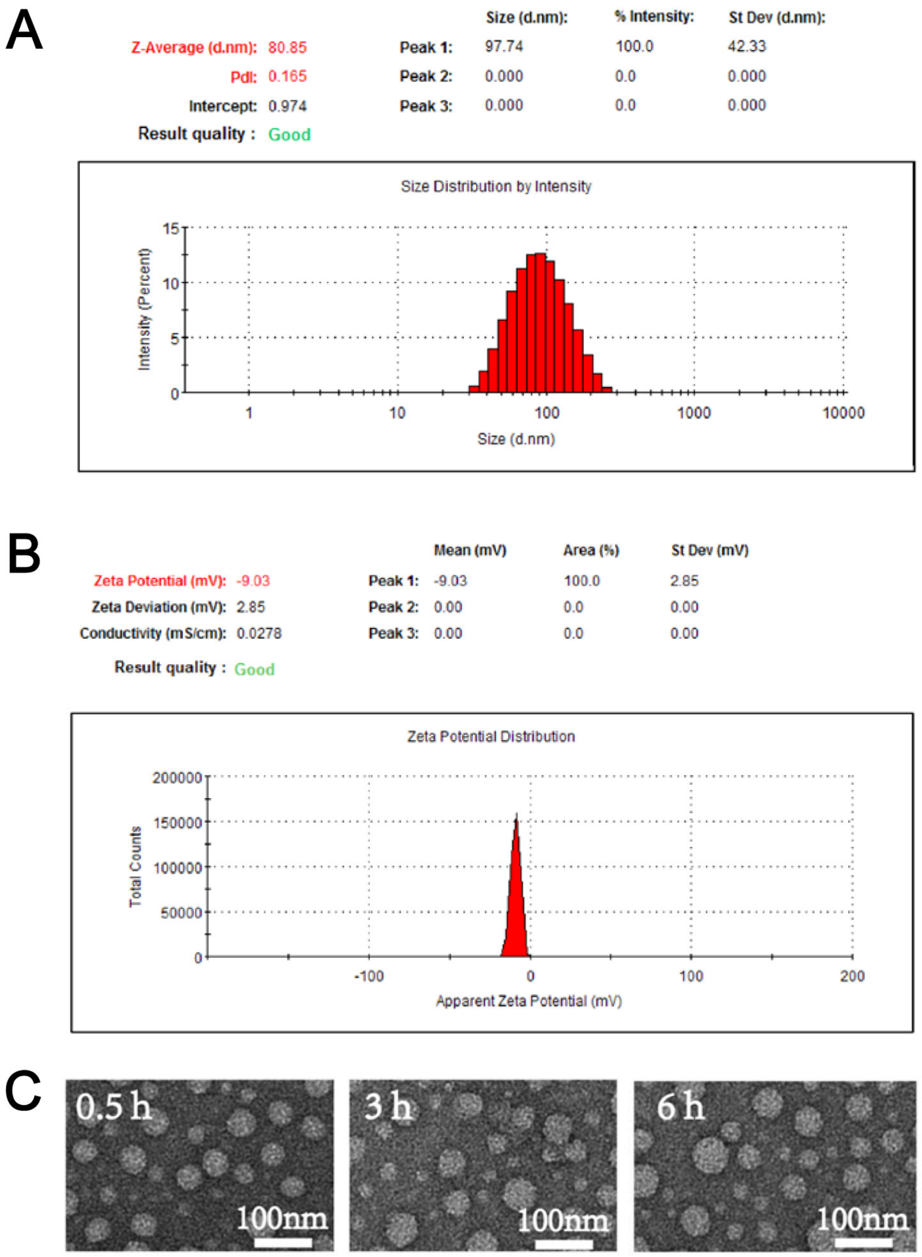

Figure S12. DLS and TEM characterization of FDPC-NPs. (A) Particle size of FDPC-NPs (B) Zeta potential of FDPC-NPs and (C) TEM images of FDPC-NPs.

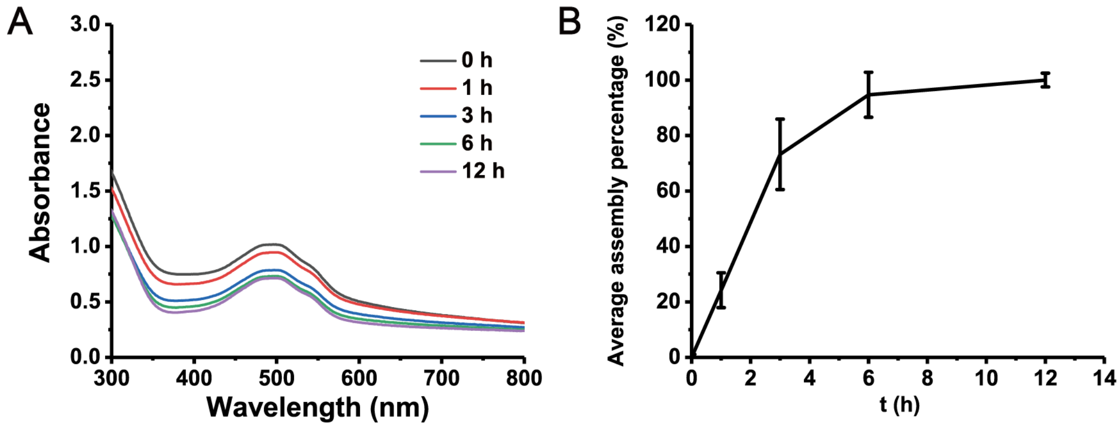

Figure S13. (A) UV-vis spectral characteristics of the FDPC-NPs self-assembly process. (B) Kinetics of FDPC-NPs self-assemble.

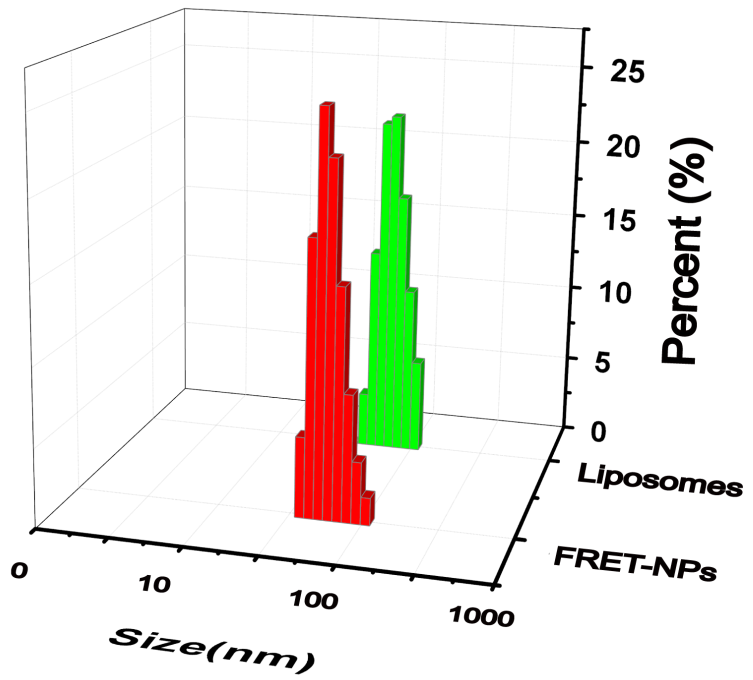

Figure S14. Particle size distribution of FRET-NPs and liposomes.

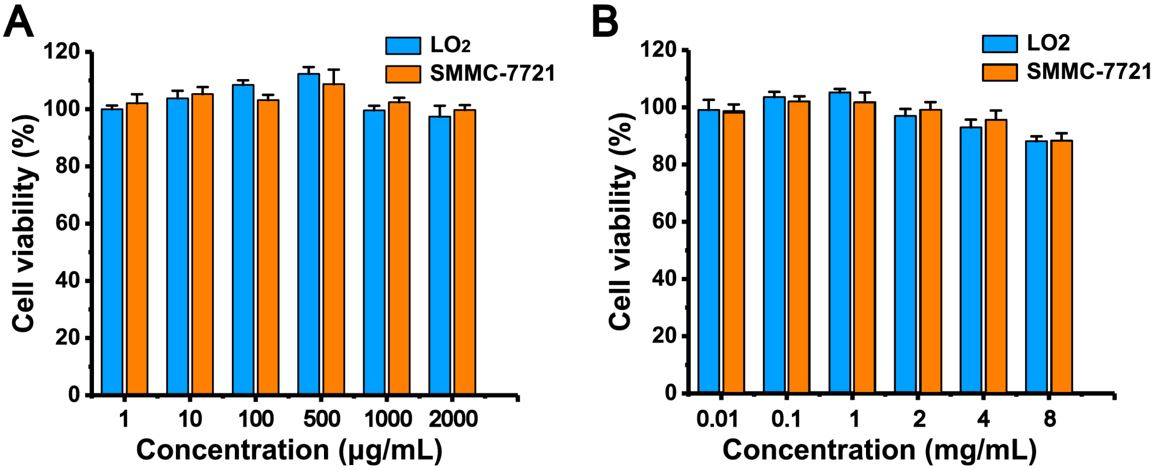

Figure S15. Relative viability of SMMC-7721 and LO2 cells treated with different concentrations of (A) peptide and (B) FPG for 24 h.

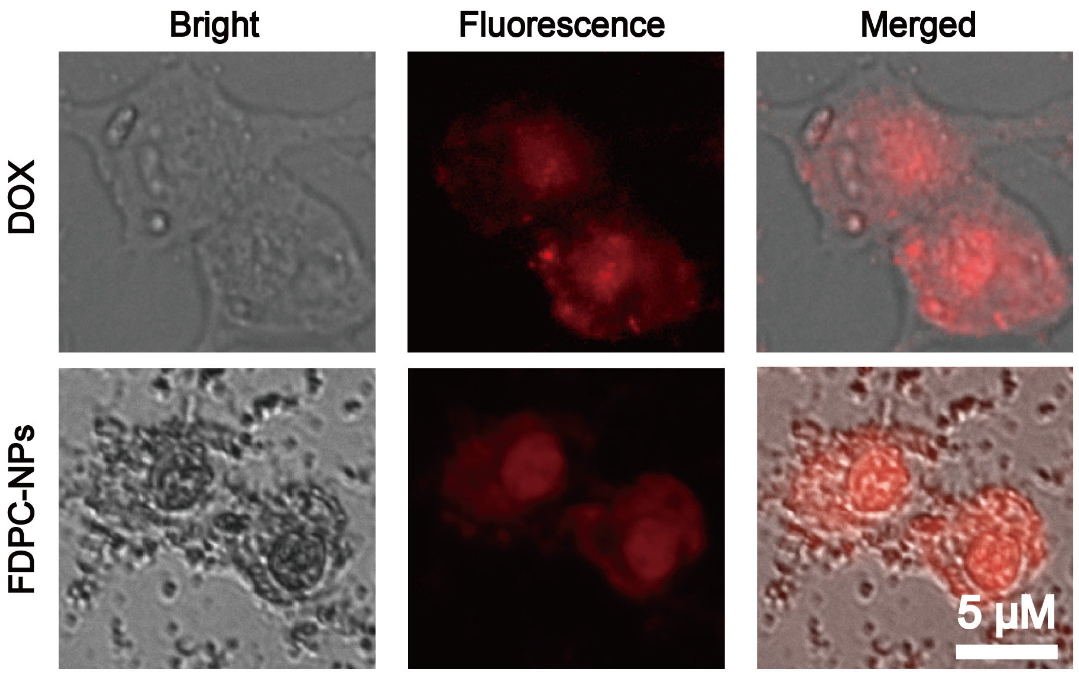

Figure S16. Fluorescence images of SMMC-7721 cells incubated with FDPC-NPs for 12 h.

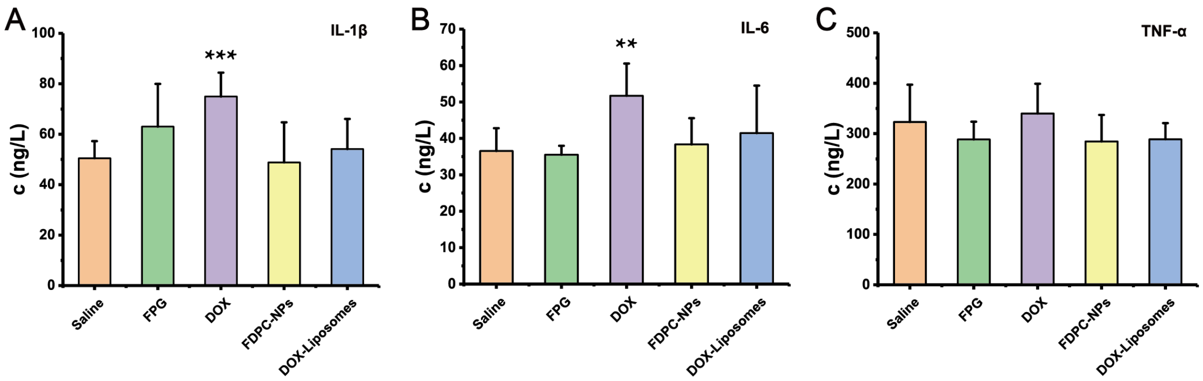

Figure S17. The concentration of IL-1β, IL-6 and TNF-α in mice sera after treat with saline control, FPG, DOX solution, FDPC-NPs or DOX liposomes at 10 mg DOX equivalent per kg (DOX solution, FDPC-NPs or DOX liposomes) and equel amount with FPDC-NPs (FPG). ** $P < 0.01$,***$P < 0.001$ compared with saline control.
